# The impact of Jinlida on blood glucose control and insulin resistance in patients with prediabetes and type 2 diabetes: a systematic review and meta-analysis of randomized controlled trials

**DOI:** 10.3389/fendo.2025.1689640

**Published:** 2025-11-17

**Authors:** Limeng Li, Qingyin Tan, Shidong Zhang, Yingxue Huang, Chuhan Xu, Shun Fan, Huanan Li, Tao Tan

**Affiliations:** 1 Tuina Department, First Teaching Hospital of Tianjin University of Traditional Chinese Medicine, Tianjin, China; 2 National Administration of Traditional Chinese Medicine Level Three Laboratory for Tuina Technique Biological Effects, Tianjin, China; 3 Department of Human Development, Teachers College, Columbia University, New York, NY, United States

**Keywords:** Jinlida, type 2 diabetes, prediabetes, systematic review, meta-analysis, glycemic control

## Abstract

**Objective:**

To evaluate the efficacy and safety of Jinlida (JLD) in improving glycemic control and insulin resistance in patients with prediabetes (PD) and type 2 diabetes mellitus (T2DM) through a meta-analysis of randomized controlled trials (RCTs).

**Methods:**

Databases including PubMed, Web of Science, Cochrane Library, Embase, Scopus, CBM, CNKI, Wanfang, and VIP were searched up to July 2025. Randomized controlled trials comparing JLD with controls were included. Pooled analyses were conducted using a random-effects model. Pre-specified subgroup analyses included health status and treatment duration. Additional analyses by baseline FBG level and age were performed to explore residual heterogeneity. Multivariable meta-regression with Knapp-Hartung adjustment further examined potential moderators (e.g., background therapy, baseline FBG level). Publication bias was assessed by funnel plots, Egger’s test, and the trim-and-fill method. Risk of bias and evidence certainty were evaluated using RoB 2.0 and GRADE.

**Results:**

A total of 20 RCTs involving 2,993 participants were included. Compared with controls, JLD significantly reduced: FBG (MD = -0.97, 95% CI: -1.40 to -0.53; *p* < 0.001), 2h-PG (MD = -1.52, 95% CI: -1.89 to -1.16; *p* < 0.001), HbA1c (MD = -0.76, 95% *CI:* -1.00 to -0.52; *p* < 0.001), HOMA-IR (MD = -0.78, 95% CI: -1.12 to -0.44, *p* < 0.001). In lipid outcomes, JLD improved: HDL-C (MD = 0.22, 95% *CI:* 0.12 to 0.32, *p* < 0.001), LDL-C (MD = -0.69, 95% CI: -1.05 to -0.33; *p* < 0.001), TC (MD = -0.57, 95% CI: -0.87 to -0.27; *p* < 0.001), TG (MD = -0.52, 95% CI: -0.72 to -0.31, *p* < 0.001). Subgroup analyses revealed that JLD produced greater glycemic improvements in shorter-duration trials and in patients with higher baseline fasting glucose (≥10 mmol/L). T2DM patients showed more pronounced reductions in HbA1c, HOMA-IR, and lipid parameters compared with PD. Additionally, JLD significantly improved HDL-C, LDL-C, TC, and TG, with lipid benefits being stronger in T2DM.

**Conclusion:**

JLD may help improve glycemic control, insulin resistance, and lipid profiles in patients with T2DM and prediabetes. Given the varying levels of certainty in the evidence across outcomes, these findings should be interpreted cautiously. Further large-scale, high-quality RCTs are needed to confirm these findings.

**Systematic review registration:**

https://www.crd.york.ac.uk/prospero/, identifier CRD420251124510.

## 1 Introduction

Type 2 diabetes mellitus (T2DM) is a chronic and progressive metabolic disorder characterized by sustained hyperglycemia that disrupts the normal metabolism of glucose, lipids, and proteins. Such metabolic disturbances accelerate cellular damage and ultimately impair the function of multiple organ systems, particularly the cardiovascular system, peripheral nerves, and vascular network (1). Recent reports indicate that the number of adult diabetes patients worldwide is as high as 589 million. It is projected that by 2050, the total number of adult diabetes patients globally will increase to 853 million (2). Prediabetes (PD) represents a transitional metabolic stage before the onset of diabetes. Individuals in this transitional phase exhibit glucose levels above the normal range yet still below the threshold for diabetes diagnosis, and these abnormalities are already linked to metabolic disturbances as well as elevated risks of cardiovascular disease, microvascular injury, cancer, and other complications (3, 4). Evidence indicates that nearly one-quarter of PD patients progress to T2DM within 3–5 years, and up to 70% eventually develop diabetes during their lifetime (5, 6). The prevalence of PD and diabetes continues to rise, not only increasing the incidence of complications but also imposing a heavy socioeconomic burden, thereby making their prevention and management an urgent global public health priority (7). Beyond hyperglycemia itself, diabetes is a leading cause of chronic kidney disease and end-stage renal disease, with many patients eventually requiring dialysis, which markedly reduces the quality of life and increases healthcare expenditures (8). Diabetic retinopathy remains a major cause of blindness worldwide (9), while diabetic neuropathy severely impairs daily functioning and substantially contributes to disability-adjusted life years (DALYs) (10). The Global Burden of Disease Study 2019 reported that the age-standardized DALY rate for T2DM was 801.5 per 100,000 population, representing a 27.6% increase compared with 1990 (11). From an economic perspective, the American Diabetes Association estimated that the annual cost of diabetes in the United States alone reached USD 412.9 billion in 2022, including direct medical expenditures and productivity losses (12). Collectively, these findings underscore that PD and diabetes are systemic diseases with a profound societal impact.

Currently, the cornerstone of T2DM and PD management includes lifestyle modifications and oral hypoglycemic agents (13). However, these approaches have notable limitations in real-world settings. Lifestyle interventions are often difficult to sustain, particularly among high-risk populations (14). Despite the availability of several pharmacological options, including metformin, sulfonylureas, and SGLT-2 inhibitors, each is accompanied by certain limitations. For instance, prolonged use of metformin may lead to gastrointestinal side effects, impaired vitamin B12 absorption, and, in rare cases, lactic acidosis (15). Sulfonylureas may cause hypoglycemia and weight gain (16), while SGLT-2 inhibitors carry an increased risk of genitourinary tract infections (17). Therefore, there is an urgent need to explore a safe adjunctive therapeutic approach. Moreover, given the progressive nature of T2DM, monotherapy with lifestyle changes or a single antidiabetic agent is often insufficient for long-term metabolic control. Many patients eventually require insulin therapy, which increases psychological and financial burdens.

Over the past decade, traditional Chinese medicine (TCM) has attracted growing attention as an adjunctive approach in diabetes management. Jinlida (JLD) is a standardized Chinese patent medicine composed of 17 herbs, such as Panax ginseng, Polygonatum sibiricum, Atractylodes lancea, Sophora flavescens, Salvia miltiorrhiza, Pueraria lobata, and Lycium barbarum. It was approved in 2005 for use alongside conventional therapy. T2DM was subsequently listed in the China National Essential Medicines List and National Reimbursement Drug List. Experimental studies indicate that JLD acts on multiple biological targets, enhancing pancreatic β-cell function, stimulating insulin secretion, preventing β-cell apoptosis, and improving insulin resistance.

Previous meta-analyses have examined the efficacy of JLD in patients with T2DM, yet their findings remain inconsistent (18, 19). These reviews were confined to individuals with established T2DM and focused mainly on a limited set of glycemic outcomes, without systematic evaluation of other metabolic indicators or safety profiles. In addition, the potential influences of participant characteristics and intervention parameters, such as age, disease stage, treatment duration, and background therapy, have not been quantitatively explored. However, such factors may substantially affect the metabolic response to JLD and determine its applicability across diverse patient groups. Considering these limitations and the publication of several new randomized controlled trials since early 2022, an updated and comprehensive synthesis is warranted. The present meta-analysis therefore evaluated both T2DM and prediabetes populations and incorporated broader clinical outcomes, including insulin resistance (HOMA-IR), lipid profiles, and adverse events. Furthermore, subgroup and meta-regression analyses were conducted to investigate how participant and intervention characteristics may influence treatment effects. This approach provides a unified analytic framework to clarify the therapeutic potential of JLD and to guide its optimized, evidence-based application in metabolic disease management.

## 2 Materials and methods

This systematic review and meta-analysis followed the PRISMA 2020 guidelines. The study protocol was prospectively registered in PROSPERO (CRD420251124510).

### 2.1 Search strategy

Two reviewers independently searched the literature published between the establishment of the databases and July 2025 in nine databases, including PubMed, Web of Science, Cochrane Library, Embase, Scopus, China Biology Medicine Database (CBM), China National Knowledge Infrastructure (CNKI), Wanfang Data, and Chinese Scientific Journal Database. At the same time, the references of relevant studies were manually retrieved. The detailed search strategy for each database was provided in Appendix 1.

### 2.2 Inclusion criteria

1. Study design: Randomized controlled trials.
2. Population: Adults who met the diagnostic criteria for PD or T2DM (20, 21).
3. Intervention measures: JLD Granules alone, or in combination with non-traditional Chinese medicine therapies, such as oral hypoglycemic agents or lifestyle interventions. Trials involving injections, decoctions, patented TCM pills, or herbal extracts were not considered TCM therapies and thus were excluded.
4. Control group: Control arms included placebo or non-TCM therapies, explicitly comprising (i) standard-of-care oral hypoglycemic agents, (ii) lifestyle intensification alone, or (iii) both.
(5) Outcomes: FBG, 2h-PG, Homeostatic Model Assessment of Insulin Resistance (HOMA-IR), HbA1c, Total Cholesterol (TC), Triglycerides (TG), Low-Density Lipoprotein Cholesterol (LDL-C), High-Density Lipoprotein Cholesterol (HDL-C).

### 2.3 Exclusion criteria

1. Non-original or incomplete studies: Abstracts, conference proceedings, reviews, case reports, letters, and secondary analyses.
2. Research on serious deficiencies in method and detail reporting.
3. Republished literature.

### 2.4 Literature selection and data extraction

The screening and data extraction processes were independently performed by two investigators (HYX and XCH). Literature duplicates were eliminated with EndNote 20.1, followed by title and abstract screening to remove non-eligible records. Full texts of potentially relevant studies were further evaluated using the prespecified eligibility criteria to determine final inclusion. Data collection covered general study information (first author, year, location), participant features (such as age, sex, disease duration, and overall health status), intervention details for the experimental group (type, frequency, length of treatment, and dosage), information on the control group, major outcomes, and adverse event reporting.

### 2.5 Quality assessment

Two researchers (LLM and TQY) independently used the Cochrane Risk of Bias Tool ROB 2.0 to evaluate the quality of these studies (22). The Cochrane methodological quality assessment tool consists of seven items, namely: random sequence generation, allocation concealment, blinding of subjects and researchers, blinding of outcome evaluators, incomplete outcome data, selective reporting, and other biases. In case of disagreement between the two researchers, a third evaluator reviewed the study to reach a consensus.

### 2.6 Statistical analysis

To ensure consistency across studies, continuous outcomes originally reported in different units were converted into a common unit before analysis. For continuous outcomes, pooled effect sizes were expressed as mean differences (MDs) with 95% confidence intervals (CIs), and for dichotomous outcomes, as risk ratios (RRs) with 95% CIs. In line with the Cochrane Handbook recommendations, when multi-arm trials were encountered, all relevant intervention groups were combined into a single intervention group and all relevant control groups into a single comparator group. All effect sizes were synthesized using a random-effects model with restricted maximum likelihood (REML) estimation. Between-study heterogeneity was evaluated using Cochran’s Q test, Higgins’ I² statistic, and 95% prediction intervals.

Sensitivity analyses were conducted using multiple approaches, including: (1) leave-one-out analysis; (2) exclusion of trials with moderate-to-high risk of bias; (3) exclusion of studies without standardized Western medicine background interventions; (4) alternative continuity corrections for sparse binary outcomes; and (5) residual diagnostics (studentized residuals, Cook’s distance, DFBETAS, hat values, and covariance ratios) to identify potential outliers and influential studies (23, 24).

The pre-specified subgroup analyses included health status (T2DM vs. PD) and treatment duration (<12 vs. ≥12 weeks). Because substantial heterogeneity remained, we conducted additional subgroup analyses by baseline FBG (<7.0, 7.0–9.9, ≥10.0 mmol/L) and age (<45, 45–59, 60–74 years). To further investigate heterogeneity, we performed multivariable random-effects meta-regression with Knapp–Hartung adjustment, incorporating background treatment, age, trial duration, baseline FBG, and health status as covariates. This approach enabled us to assess the moderating effects of multiple clinical and methodological factors and to quantify the proportion of variance explained (R²). Publication bias was examined by funnel plot symmetry and Egger’s regression test when ten or more randomized controlled trials (RCTs) were available for an outcome. If publication bias is detected, the trim-and-fill method will be used to adjust for it. All statistical analyses were performed using R software (Version 4.4.3).

### 2.7 Evidence quality evaluation

This study applied the GRADE (Grading of Recommendations, Assessment, Development and Evaluation) approach to evaluate the quality of evidence for primary outcomes (25), considering five domains: risk of bias, inconsistency, indirectness, imprecision, and publication bias. Evidence was rated as high, moderate, low, or very low. Two reviewers (LLM and FS) independently conducted the assessment, with discrepancies resolved through discussion or by consulting a third reviewer. This rigorous process ensured the objectivity and reliability of the evaluation.

## 3 Results

### 3.1 Study selection

**Figure 1:**
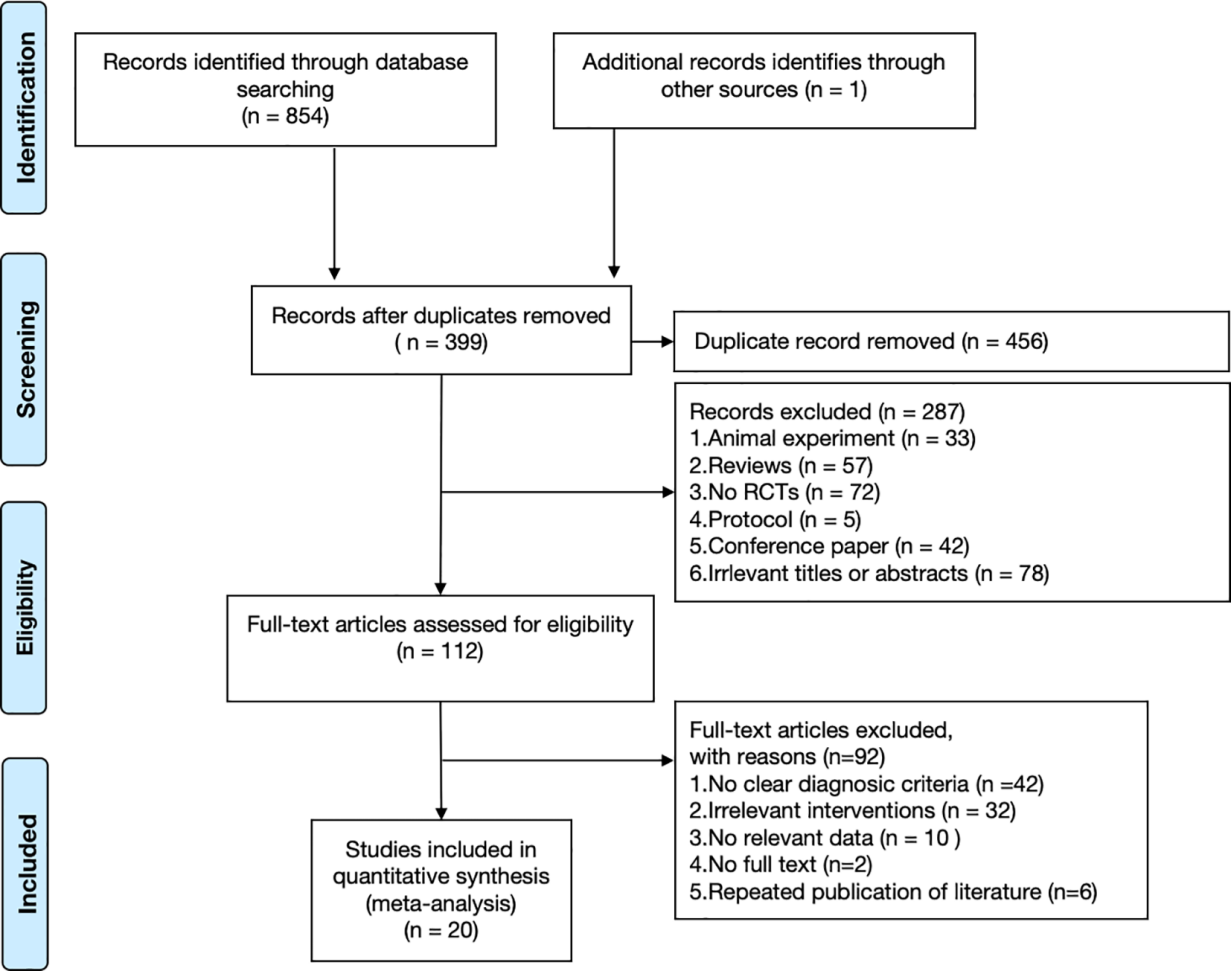
Flow diagram of study selection process.

A total of 855 relevant studies were initially retrieved. After removing duplicates, 399 records remained for title and abstract screening. After screening titles and abstracts, 287 records were excluded. Subsequently, 112 full-text articles were assessed for eligibility, and 92 were excluded for not meeting the inclusion criteria. Ultimately, 20 RCTs were included in the present meta-analysis (26–45). The literature screening process and results are presented in **Figure 1**.

### 3.2 Study characteristics

A total of 20 trials involving 2,993 subjects were included in this meta-analysis. These RCTs were published between 2015 and 2024. All studies were conducted in China. Of these, six were published in English and fourteen in Chinese. In two studies, the participants had PD, while in the remaining studies, participants had T2DM. Sample sizes ranged from 58 to 885. The duration of the intervention ranged from 4 weeks to 16 weeks. The average age of participants ranged from (44.12 ± 4.31) to (69.6 ± 2.7) years. The characteristics of the included trials are summarized in **Table 1**.

**Table 1:** Baseline characteristics of the included studies.

Studies	Year	Country	Group	Intervention	Number of subjects	Age (years)	Sex (Male/Female)	Course of disease	Participant	Trial duration	Dose
Cai, J (26).	2023	China	IG	JLD + Metformin, atorvastatin	66	47.45 ± 10.48	36 (30)	/	T2DM+MAFLD	12w	9mg, tid
			CG	Metformin, atorvastatin	66	48.09 ± 10.37	35 (31)	/			
Fan, H. J (27).	2021	China	IG	JLD + Nateglinide Tablet	64	51.48 ± 5.29	37 (27)	3.24 ± 1.37	T2DM	8w	9mg, tid
			CG	Nateglinide Tablet	64	53.27 ± 4.29	38 (26)	3.52 ± 1.48			
Lian, F (28).	2015	China	IG	JLD + Metformin	92	55.18 ± 9.13	53 (39)	5.68 ± 3.58	T2DM	12w	9g, tid
			CG	Placebo + Metformin	94	55.81 ± 9.93	54 (40)	6.18 ± 4.10			
Pan, J (29).	2021	China	IG	A:JLD + placebo tablets B:placebo tablets + placebo granules	A:34 B:33	A:51.59 ± 10.31 B:54.57 ± 10.54	A:25 (9) B:22 (11)	/	T2DM	16w	9g, tid
			CG	C:placebo tablets + placebo granules D:metformin tablets + placebo granules	C:35 D:36	C:56.53 ± 9.18 D:56.00 ± 9.47	C:23 (12) D:24 (12)	/			
Shi, Y. L (30).	2016	China	IG	JLD	32	47.1 ± 7.1	17 (15)	/	IGT	12w	9g, tid
			CG	Standard diet control and exercise therapy	29	49.9 ± 7.2	14 (15)	/			
Ji, H (31).	2024	China	IG	JLD	442	52.26 ± 10.10	218 (224)	/	IGT	24m	9g, tid
			CG	placebo	443	52.88 ± 10.57	204 (239)	/			
Hou, L. P (32).	2023	China	IG	JLD+ Semaglutide + Metformin	50	66.71 ± 6.61	29 (21)	9.38 ± 4.78 (year)	T2DM+CHD	12w	9g, tid
			CG	Semaglutide + Metformin	50	67.24 ± 5.57	28 (22)	8.79 ± 3.11 (year)			
Fu, Y. F (33).	2019	China	IG	JLD+ Metformin + Liraglutide Injection	97	45.09 ± 3.52	53 (44)	8.19 ± 2.25 (year)	T2DM	12w	9g, tid
			CG	Metformin + Liraglutide Injection	96	44.12 ± 4.31	53 (44)	8.24 ± 2.38 (year)			
Liu, H (34).	2018	China	IG	JLD + Glipizide Sustained Release Capsules	60	45.94 ± 9.86	33 (27)	30.83 ± 8.56(months)	T2DM	8w	9g, tid
			CG	Glipizide Sustained Release Capsules	60	46.73 ± 7.18	32 (29)	32.15 ± 7.09(months)			
Liu, L. K (35).	2017	China	IG	JLD + Metformin	60	69.1 ± 8.2	29 (31)	/	T2DM	8w	9g, tid
			CG	Metformin	60	68.6 ± 7.1	34 (26)	/			
Zhou, X. H (36).	2020	China	IG	JLD+GLP-1RA	46	55.74 ± 1.53	25 (21)	8.62 ± 1.89 (year)	T2DM	8w	9g, tid
			CG	GLP-1RA	46	8.62 ± 1.89	26 (20)	8.13 ± 1.37 (year)			
Han, L (37).	2024	China	IG	JLD + dapagliflozin	71	69.3 ± 2.5	41 (30)	7.8 ± 1.3 (year)	T2DM	12w	9g, tid
			CG	dapagliflozin	71	69.6 ± 2.7	40 (31)	7.5 ± 1.6 (year)			
Zhao, Z. L (38).	2022	China	IG	JLD + liraglutide injection	30	56.83 ± 7.12	20 (10)		T2DM	12w	9g, tid
			CG	liraglutide injection	30	57.01 ± 6.57	17 (13)				
Zhao, S. Y (39).	2022	China	IG	JLD + Metformin	35	51.57 ± 5.14	15 (20)	4.54 ± 2.07 (year)	T2DM+MAFLD	12w	9g, tid
			CG	Metformin	35	51.08 ± 6.34	16 (19)	4.91 ± 2.47 (year)			
Cai, J.(a) (40)	2022	China	IG	JLD + metformin	30	47.3 ± 7.4	14 (16)	5.01 ± 2.36 (year)	T2DM	12w	9g, tid
			CG	metformin	28	47.3 ± 8.9	13 (15)	5.05 ± 2.49 (year)			
Cai, J.(b) (41)	2022	China	IG	JLD + liraglutide injection	40	51.10 ± 7.74	24 (16)	4.62 ± 2.45 (year)	T2DM+MAFLD	12w	9g, tid
			IG	liraglutide injection	40	51.47 ± 9.55	26 (14)	4.22 ± 2.75 (year)			
Hu, X. B (42).	2022	China	CG	JLD + sitagliptin	45	66.5 ± 6.8	23 (22)	6.1 ± 2.5 (year)	T2DM	12w	9g, tid
			IG	sitagliptin	45	66.8 ± 6.2	21 (24)	5.9 ± 2.2 (year)			
Huang, J. C (43).	2020	China	CG	JLD + Insulin Aspart 30 Injection	49	51.04 ± 8.31	26 (23)	3.62 ± 1.66 (year)	T2DM	12w	9g, tid
			IG	Insulin Aspart 30 Injection	49	54.49 ± 6.15	24 (25)	3.49 ± 1.87 (year)			
Jiang, W (44).	2020	China	CG	JLD + Dapagliflozin Tablets	42	67.3 ± 4.1	26 (16)	7.4 ± 1.9 (year)	T2DM	12w	9g, tid
			IG	Dapagliflozin Tablets	42	66.8 ± 3.7	23 (19)	7.0 ± 2.2 (year)			
Zhao,J.D (45).	2017	China	CG	JLD +Metformin	78	51.76 ± 8.88	46 (32)	9.97 ± 3.4 (year)	T2DM	12w	9g, tid
			IG	Metformin + placebo tablets	78	49.44 ± 10.93	50 (28)	9.89 ± 3.39 (year)			

IG, intervention group; CG, control group; JLD, JinLida; T2DM, type 2 diabetes mellitus; MAFLD, Metabolic Dysfunction-Associated Fatty Liver Disease; CHD, Coronary Heart Disease; DPN, Diabetic Peripheral Neuropathy; IGT, Impaired glucose tolerance; IFG, impaired fasting glucose; w, weeks; m, months; tid, three times a day, (a)Different studies by the same investigator, Study 1, (b) Different studies by the same investigator, Study 2.

### 3.3 Risk of bias

**Figure 2:**
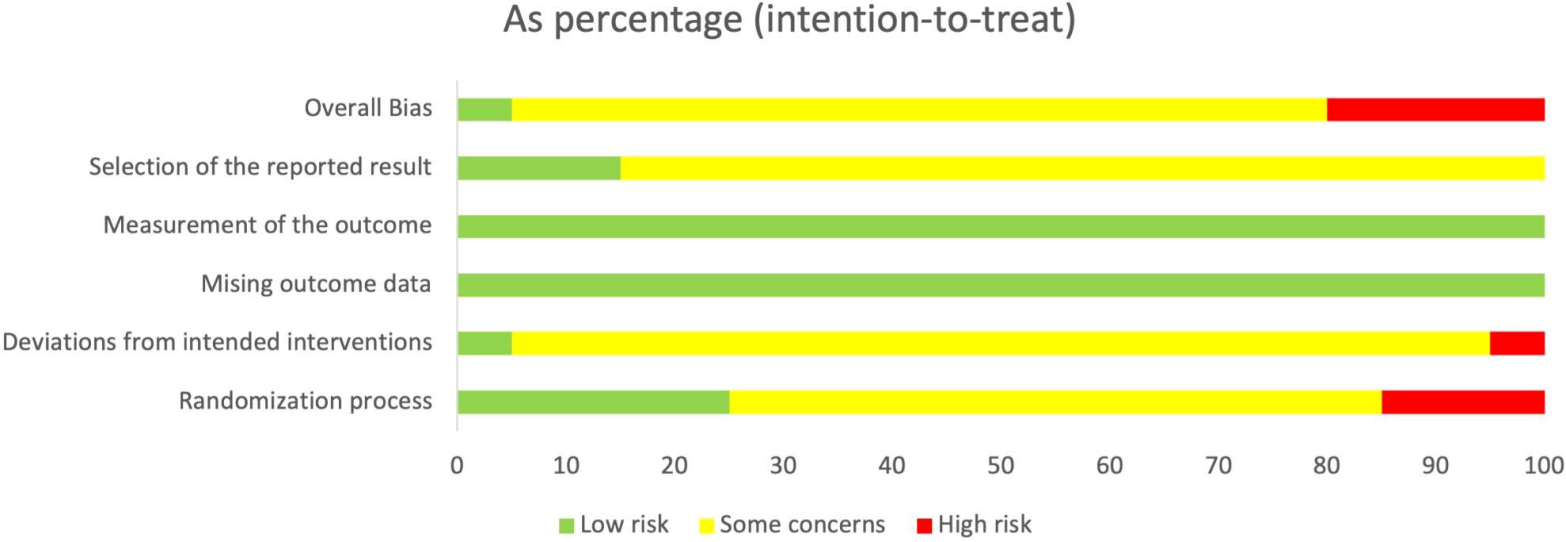
The graph of risk of bias summary.

**Figure 3:**
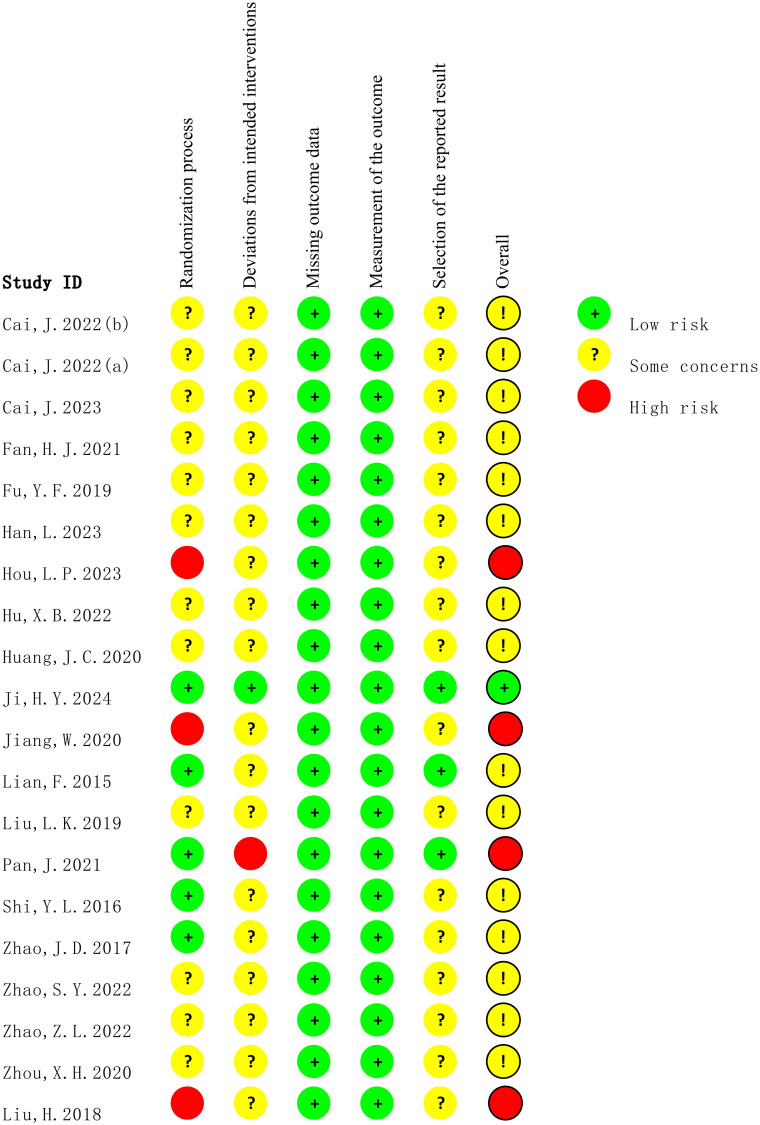
Risk of bias assessment across domains.

The methodological quality of all included studies was assessed using the ROB 2.0. As shown in **Figure 2** and **Figure 3**, most studies reported randomization procedures but were rated as having “some concerns” in the domain of Randomization process due to insufficient description of allocation concealment. Three studies (32, 34, 35) were judged to be at “high risk” in this domain. In the domain of deviations from intended interventions, although most studies did not explicitly apply intention-to-treat (ITT) analysis, the proportion of participants who did not adhere to their assigned interventions was less than 5%, which was unlikely to substantially affect the results; therefore, these studies were rated as “low risk” or “some concerns.” However, the study by Pan, J (29). relied on per-protocol (PP) analysis rather than ITT, with a dropout rate of 12.7%, which may have introduced bias, and was thus rated as “high risk.” For outcome measurement, all studies were rated as “low risk,” since the primary outcomes were objectively measured using standardized blood glucose tests. In the domain of selection of the reported result, most studies were judged as having “some concerns,” primarily because they did not clarify whether a pre-specified analysis plan was finalized before unblinding. Overall, most studies were judged to have “some concerns,” and four studies were rated as “high risk” in at least one domain.

### 3.4 Meta-analysis

#### 3.4.1 Effect of JLD on FBG

**Figure 4:**
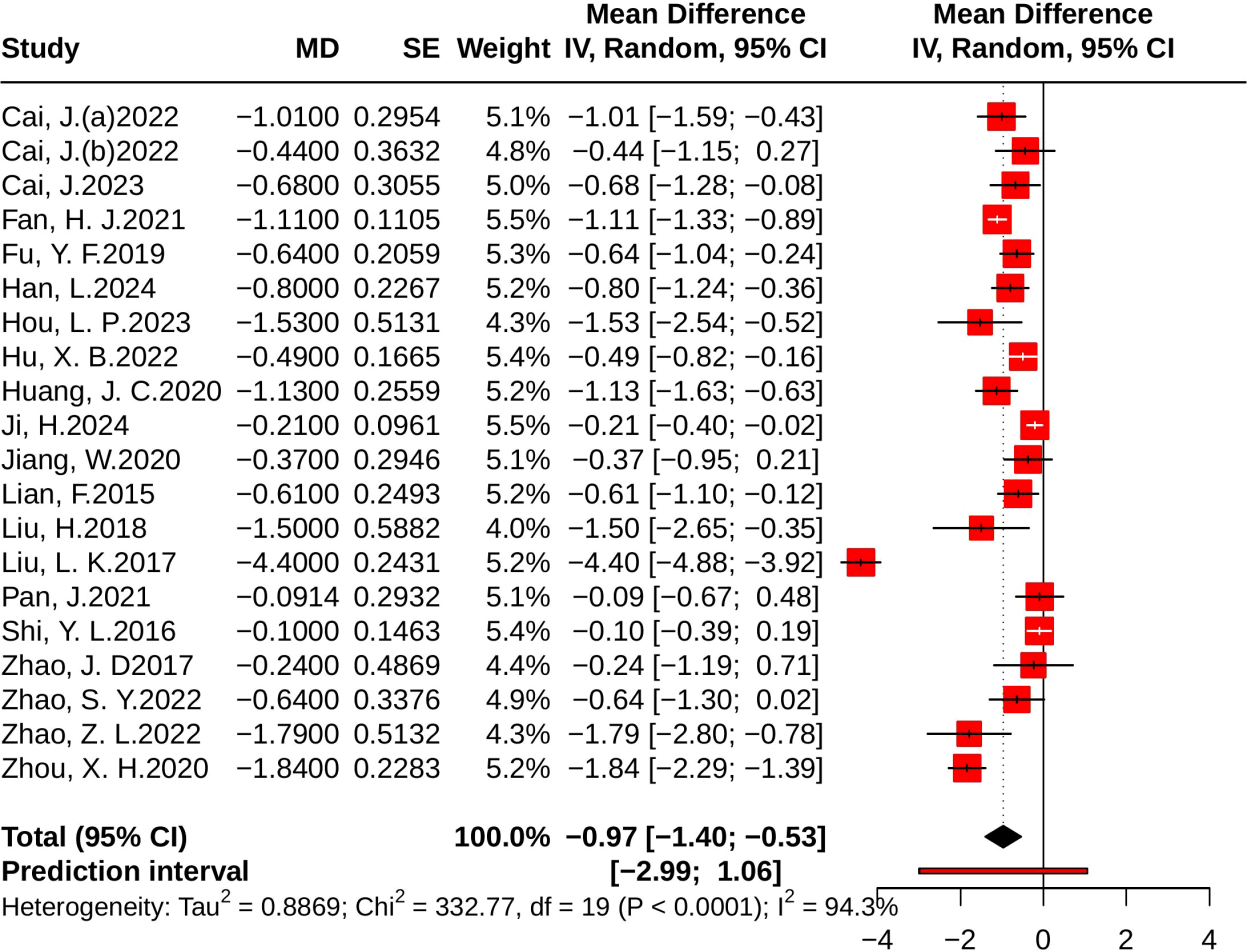
Forest plot of the effect of JLD on FBG. Study-specific mean differences (MDs) are shown as squares, with areas proportional to study weights. Horizontal bars depict 95% confidence intervals (CIs), and the pooled estimate is displayed as a diamond derived from a random-effects model. The vertical reference line at 0 indicates no overall effect.

A total of 20 studies with 2,993 participants (1,497 in the control group and 1,496 in the treatment group) evaluated the effect of JLD on FBG. Heterogeneity analysis indicated substantial variability among studies (*P* < 0.001, *I²* = 94.3%, 95% PI [-2.99, 1.06]). The pooled results showed that JLD significantly reduced FBG compared with the control group (MD = –0.97, 95% CI: –1.40 to –0.53; *p* < 0.001; **Figure 4**).

Subgroup analyses revealed greater reductions in FBG in trials lasting <12 weeks (MD = -2.23, 95% CI: -3.71 to -0.74) compared with those lasting ≥12 weeks (MD = -0.58, 95% CI: -0.78 to -0.39), with the difference significant after Holm correction (*p for interaction* = 0.0012, P_Holm = 0.0037, R² = 45.63%). Baseline FBG also modified the treatment effect: participants with baseline FBG ≥10.0 mmol/L showed the largest reduction (MD = -2.62, 95% CI: -4.48 to -0.76), those with 7.0–9.9 mmol/L had a moderate reduction (MD = -0.79, 95% CI: -1.02 to -0.55), while those with baseline <7.0 mmol/L showed only a small but statistically significant decrease (MD = -0.18, 95% CI: -0.33 to -0.02; *p* for interaction = 0.0005, *P*_Holm = 0.0020, R² = 62.61%). No significant subgroup effects were found for disease status (T2DM vs. PD; *p* = 0.201, *P*_Holm = 0.401, R² = 4.10%) or age (*p* = 0.366, *P*_Holm = 0.401, R² = 0.51%) (**Supplementary Table 1**).

#### 3.4.2 Effect of JLD on 2h-PG

**Figure 5:**
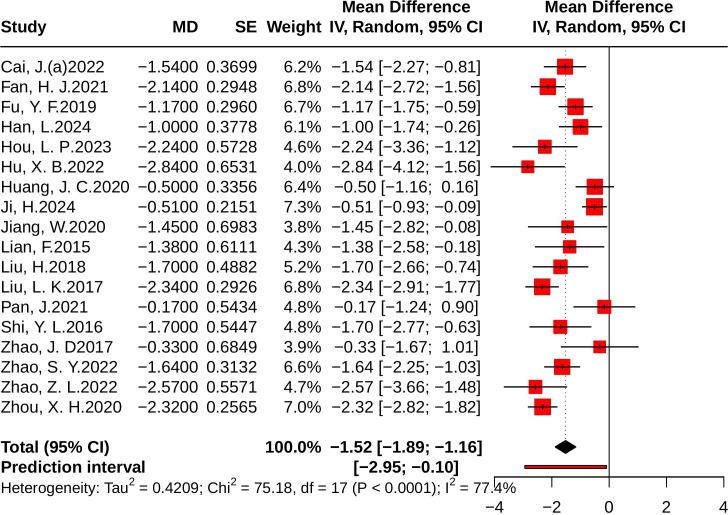
Forest plot of the effect of JLD on 2h-PG. Each square reflects the MD for an individual trial, scaled to its weight in the meta-analysis. The line across each square represents the 95% CI. The diamond at the bottom summarizes the pooled MD under a random-effects model, with the line at 0 marking the null effect.

A total of 18 studies evaluated 2h-PG, with a combined sample size of 2,781 participants, including 1,391 in the control group and 1,390 in the treatment group. Heterogeneity testing revealed moderate heterogeneity among the studies (*p * < 0.001, *I^2^* = 77.4%, 95% *PI* [-2.95, -0.10]). The meta-analysis showed that JLD significantly reduced 2h-PG levels (MD = -1.52, 95% CI: -1.89 to -1.16; *p* < 0.001), as shown in **Figure 5**.

For 2h-PG, treatment duration was again a significant moderator. Trials with <12 weeks of follow-up showed larger reductions (MD = -2.22, 95% CI: -2.52 to -1.92) compared with ≥12 weeks (MD = -1.30, 95% CI: -1.69 to -0.90), although this effect lost significance after Holm correction (*p* for interaction = 0.025, *P*_Holm = 0.101, R² = 44.53%). Baseline glucose also influenced results: participants with ≥10.0 mmol/L showed the largest benefit (MD = -2.24, 95% CI: -2.69 to -1.79), followed by those with 7.0-9.9 mmol/L (MD = -1.46, 95% CI: -1.87 to -1.04), while <7.0 mmol/L showed smaller and non-significant effects (MD = -1.00, 95% CI: -2.15 to 0.15; *p* for interaction = 0.173, *P*_Holm = 0.519, R² = 19.34%). Subgroup effects by disease status (PD vs. T2DM) and age were not statistically significant (all *P*_Holm > 0.05). Notably, the similarity between the PD subgroup and the <7.0 mmol/L subgroup likely reflects the substantial overlap of patient characteristics. Detailed results are presented in **Supplementary Table 2**.

#### 3.4.3 Effect of JLD on HbA1c

**Figure 6:**
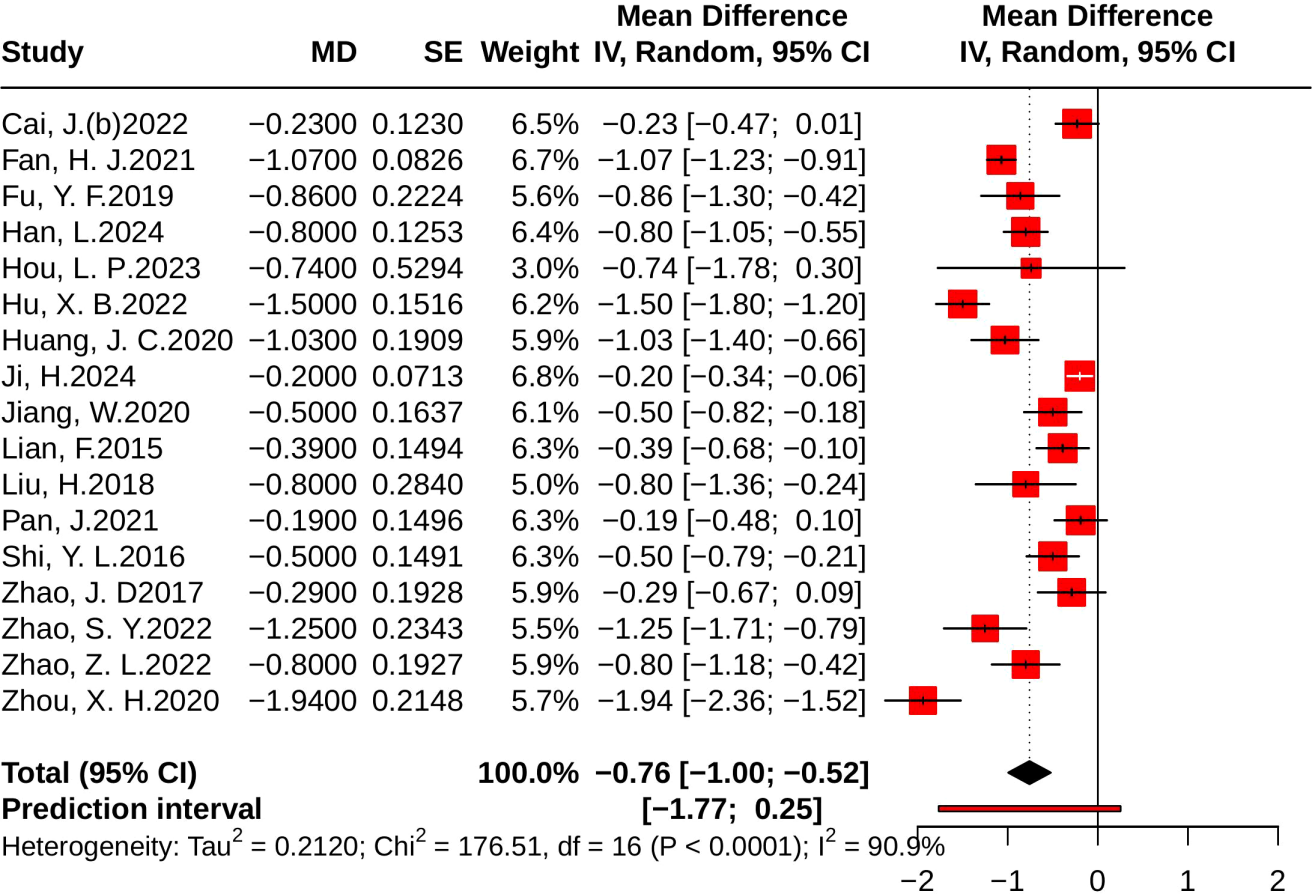
Forest plot of the effect of JLD on HbA1c. Squares denote individual study estimates (MDs) weighted by sample size contribution, with accompanying 95% CIs as horizontal whiskers. The overall random-effects estimate is presented as a diamond, and the solid vertical line at 0 represents no difference between groups.

A total of 17 studies evaluated HbA1c, involving 2,683 participants—1,343 in the control group and 1,340 in the treatment group. Heterogeneity analysis revealed substantial heterogeneity among the included studies (*p * < 0.001, *I^2^* = 90.9%, 95% PI [-1.77, 0.25]). The results of the meta-analysis showed that compared with the control group, JLD could significantly reduce 2h-PG levels (MD = -0.76, 95% CI: -1.00 to -0.52; *p* < 0.001), as illustrated in **Figure 6**.

Subgroup analyses indicated that shorter trials (<12 weeks) achieved greater reductions in HbA1c (MD = -1.21, 95% CI: -1.68 to -0.75). However, this difference did not remain statistically significant after Holm–Bonferroni correction (*p* for interaction = 0.029, *P*_Holm = 0.116, R² = 27%). Regarding baseline glucose, patients with ≥10.0 mmol/L showed a significant reduction (MD = -0.80, 95% CI: -1.11 to -0.49), but subgroup differences were not statistically significant (*p for interaction* = 0.433, *P*_Holm = 0.866, R² = 0%). No significant subgroup effects were observed for disease status (*p* = 0.187, *P*_Holm = 0.562, R² = 6.2%) or age (*p* = 0.796, *P*_Holm = 0.866, R² = 0%), as shown in **Supplementary Table 3**.

#### 3.4.4 Effect of JLD on HOMA-IR

**Figure 7:**
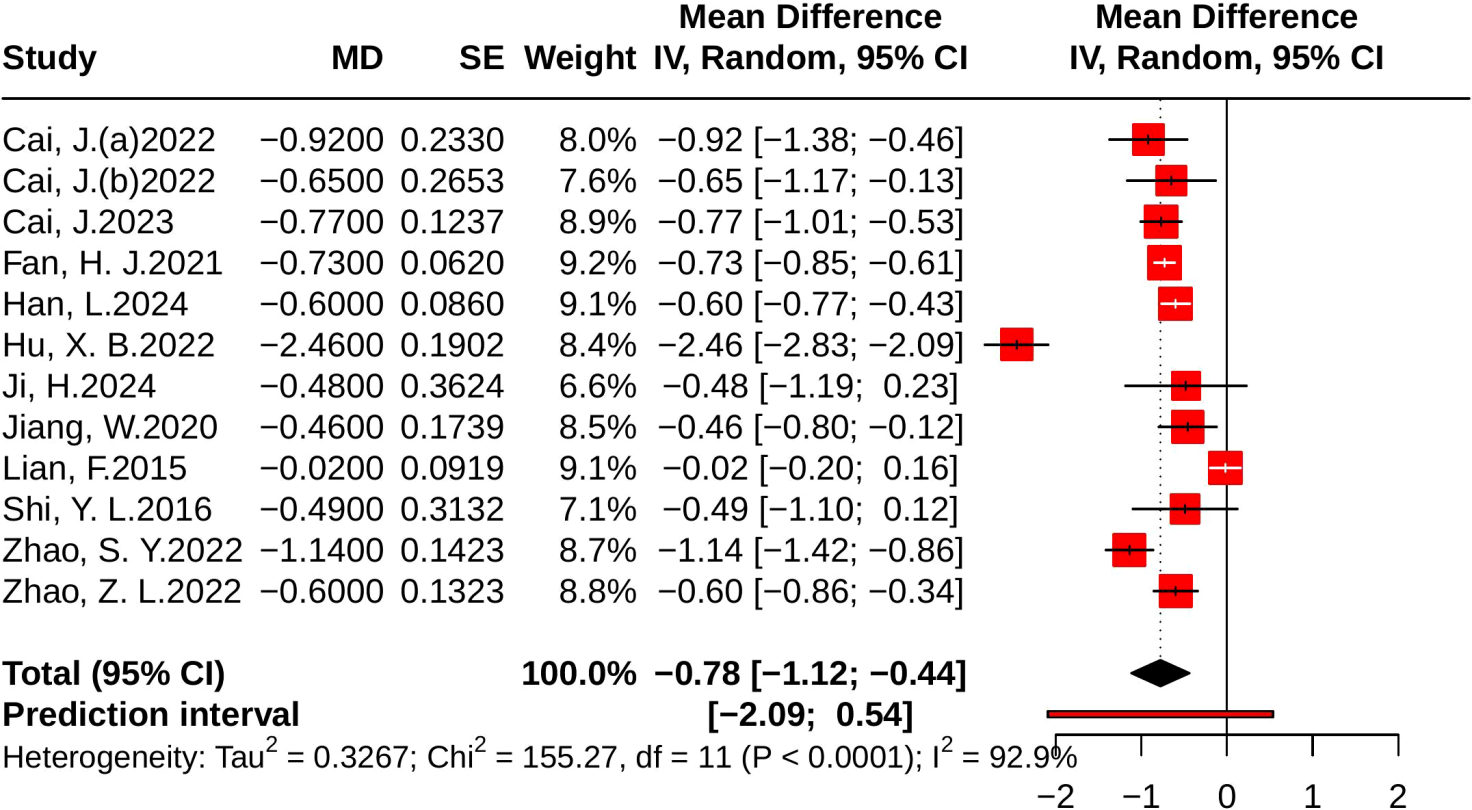
Forest plot of the effect of JLD on HOMA-IR. Each study’s MD is indicated by a square, with size corresponding to analytic weight. Horizontal lines display 95% CIs, and the pooled random-effects estimate is shown as a diamond. The vertical axis at 0 indicates the line of no effect.

Twelve studies evaluated HOMA-IR, including 989 participants in the control group and 983 in the treatment group. Heterogeneity analysis revealed substantial heterogeneity (*p* < 0.001, *I²* = 92.9%, 95% PI [-2.09, 0.54]). The meta-analysis demonstrated that JLD significantly reduced HOMA-IR compared with control (MD = -0.78, 95% CI: -1.12 to -0.44; *p* < 0.001), as illustrated in **Figure 7**.

Subgroup analyses showed no significant differences in treatment effect across trial duration (*p* for interaction = 0.934, *P*_Holm = 1.000, R² = 0%), disease status (*p* = 0.522, *P*_Holm = 1.000, R² = 0%), baseline glucose level (*p* = 0.763, *P*_Holm = 1.000, R² = 0%), or age (*p* = 0.215, *P*_Holm = 1.000, R² = 5.28%). Detailed results are presented in **Supplementary Table 4**.

#### 3.4.5 Effect of JLD on HDL-C

**Figure 8:**
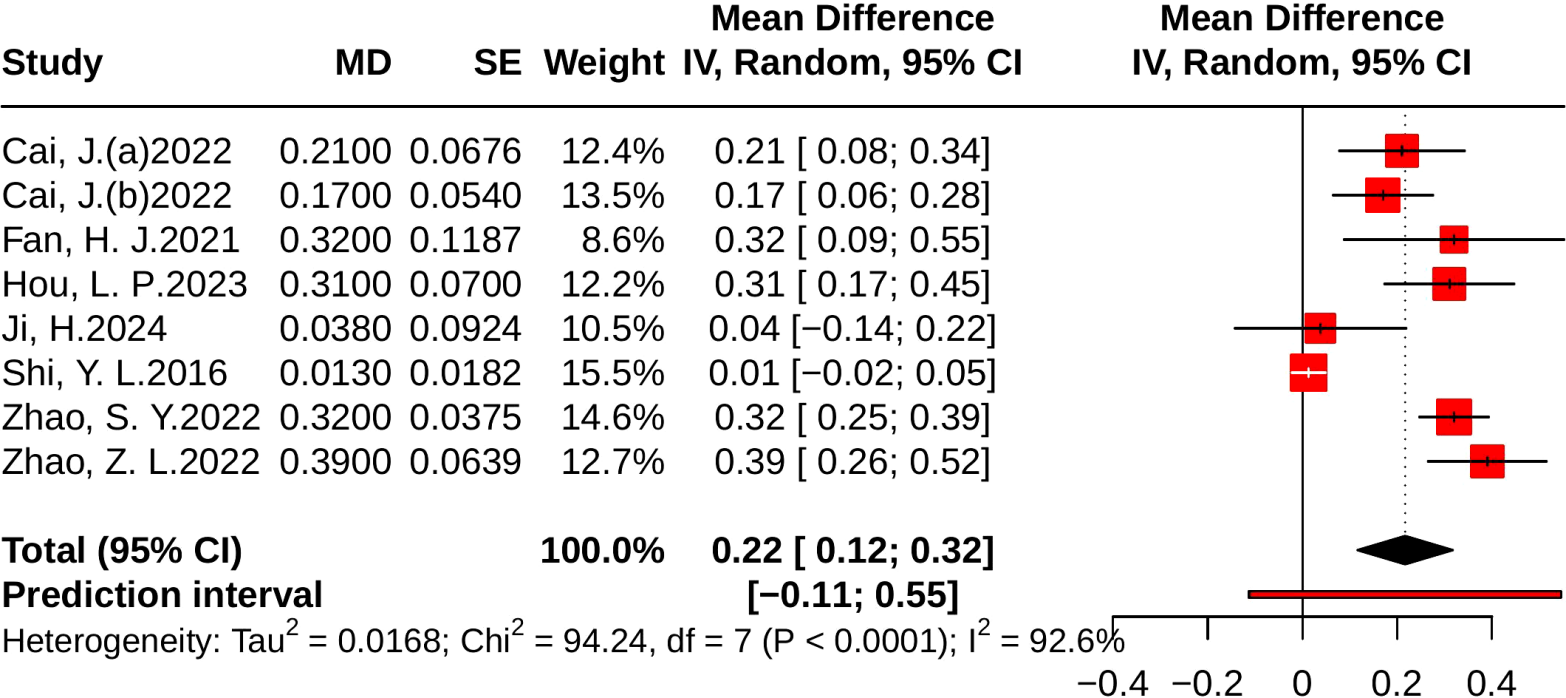
Forest plots of the effect of JLD on HDL-C. Squares reflect study-specific MDs, scaled by weight. The horizontal span of each square shows the 95% CI, while the overall pooled MD from a random-effects model is represented by a diamond. The reference line at 0 denotes no observed effect.

Eight studies evaluated HDL-C, including 1,442 participants (723 in the control group and 719 in the treatment group). Heterogeneity analysis revealed substantial between-study heterogeneity (*p* < 0.001, *I²* = 92.6%, 95% PI [-0.11, 0.55]). The meta-analysis demonstrated that JLD significantly increased HDL-C levels compared with the control group (MD = 0.22, 95% CI: 0.12 to 0.32; *p* < 0.001), as shown in **Figure 8**.

Subgroup analyses indicated greater improvements in T2DM patients (MD = 0.28, 95% CI: 0.21 to 0.35; *p* for interaction = 0.003, *P*_Holm = 0.012, R² = 84.2%) and in participants with baseline glucose ≥10.0 mmol/L (MD = 0.39, 95% CI: 0.26 to 0.52; *p* for interaction = 0.005, *P*_Holm = 0.015, R² = 89.1%). No significant subgroup effects were observed for trial duration (*p* = 0.564, *P*_Holm = 1.000, R² = 0%) or age (*p* = 0.528, P_Holm = 1.000, R² = 0%). Detailed results are presented in **Supplementary Table 5**.

#### 3.4.6 Effect of JLD on LDL-C

**Figure 9:**
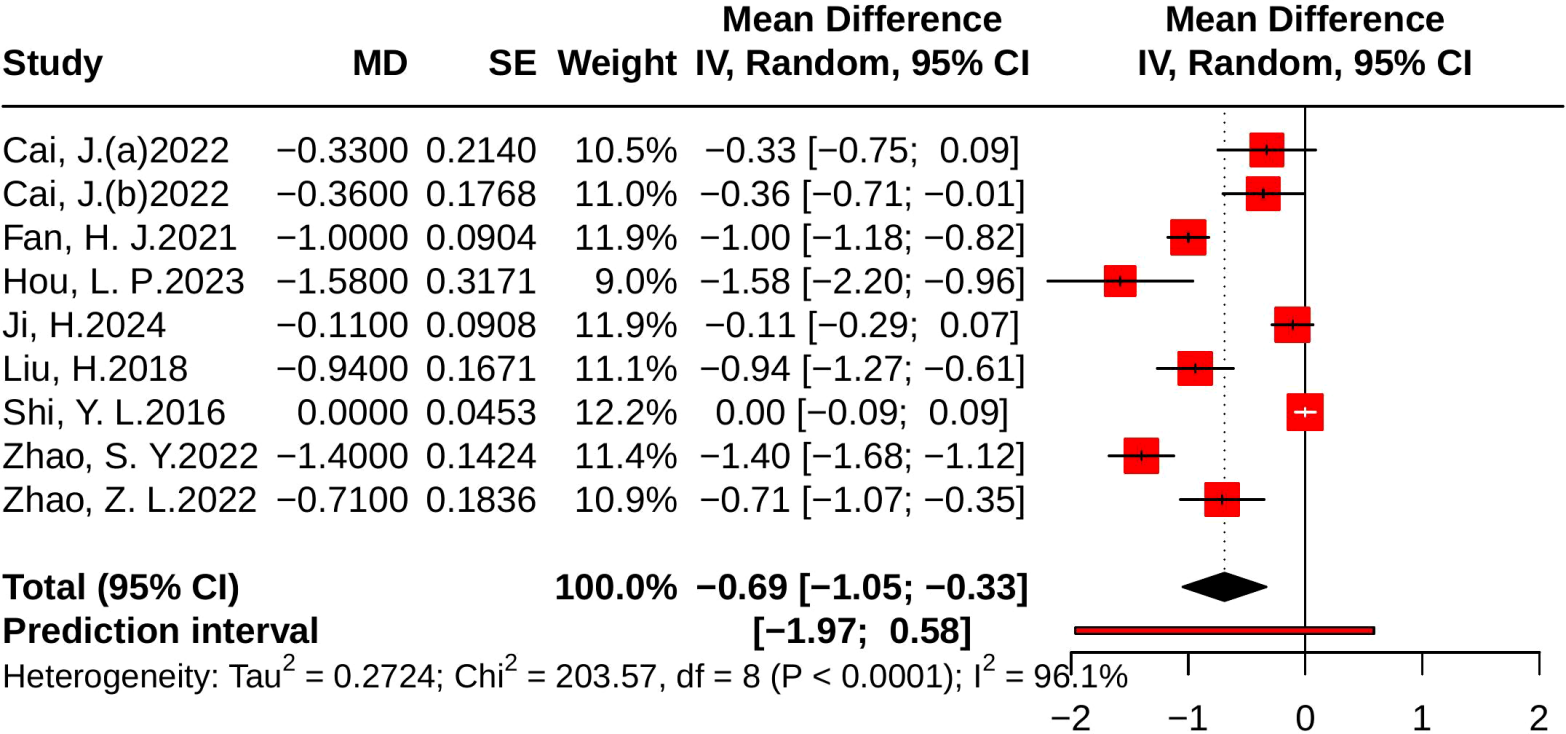
Forest plots of the effect of JLD on LDL-C. Each square corresponds to a trial estimate (MD), with size indicating relative weight. Horizontal lines indicate 95% CIs. The pooled estimate is summarized by a diamond under a random-effects model, with 0 as the null line.

Nine studies evaluated LDL-C, including 1,562 participants (779 in the control group and 783 in the treatment group). Heterogeneity analysis indicated substantial between-study variability (*p* < 0.001, *I²* = 96.1%, 95% PI [-1.97, 0.58]). The meta-analysis demonstrated that JLD significantly reduced LDL-C levels compared with the control group (MD = -0.69, 95% CI: -1.05 to -0.33; *p* < 0.001), as shown in **Figure 9**.

Subgroup analyses suggested greater LDL-C reductions in T2DM patients (MD = -0.89, 95% CI: -1.22 to -0.56; *p* for interaction = 0.033, *P*_Holm = 0.133, R² = 52.4%). By baseline glucose level, larger benefits were observed in participants with ≥10.0 mmol/L or 7.0–9.9 mmol/L, although the subgroup difference was not statistically significant (*p* = 0.128, P_Holm = 0.385, R² = 41.4%). No significant subgroup effects were found for trial duration (*p* = 0.442, *P*_Holm = 0.442, R² = 0%) or age (*p* = 0.143, P_Holm = 0.385, R² = 16.1%). Detailed results are provided in **Supplementary Table 6**.

#### 3.4.7 Effect of JLD on TC

**Figure 10:**
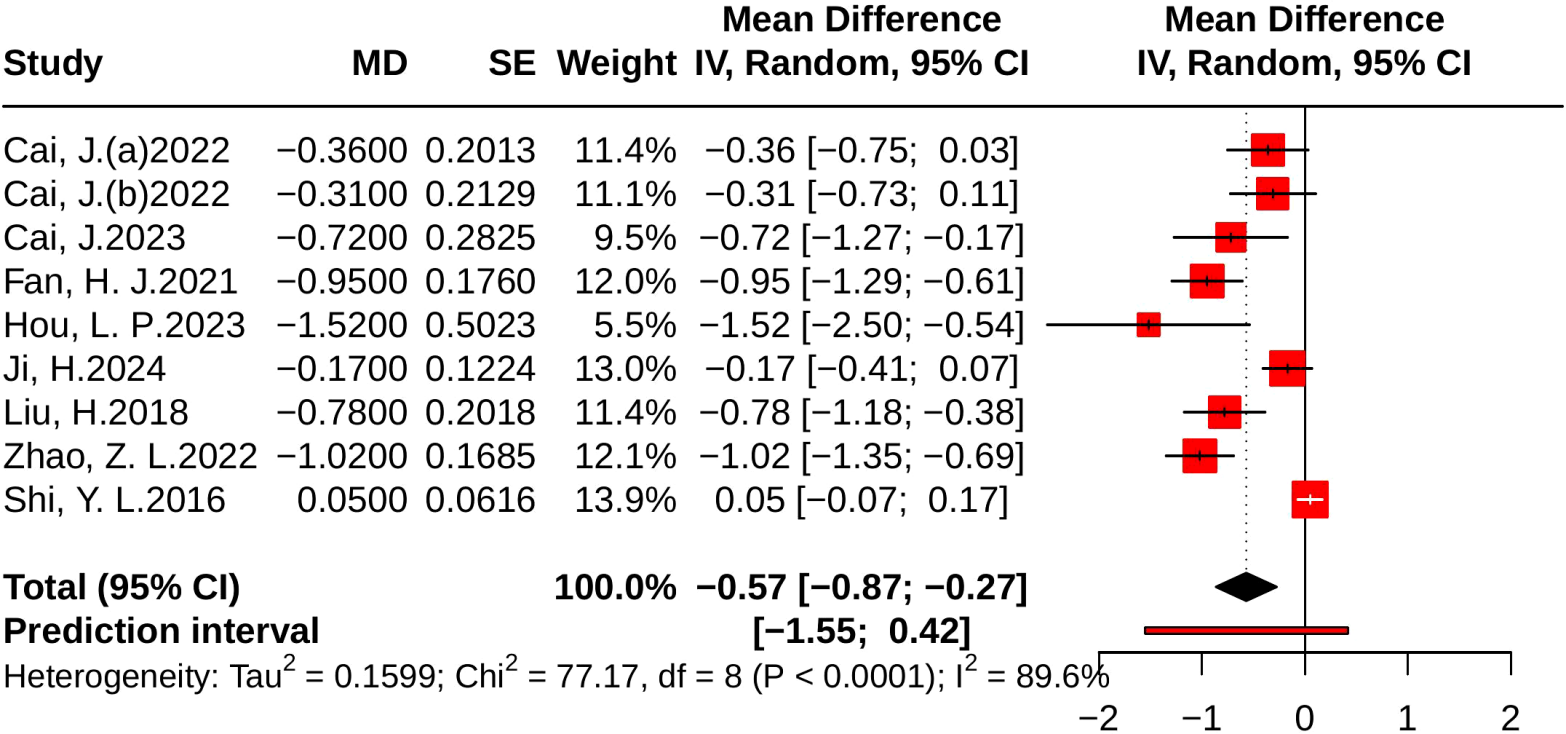
Forest plots of the effect of JLD on TC. Squares show the MDs from individual studies proportional to weight, with horizontal lines indicating 95% CIs. The pooled result is depicted as a diamond derived from the random-effects model, with the vertical line at 0 signifying no effect.

Nine studies evaluated TC, including 810 in the control group and 814 in the treatment group. Heterogeneity analysis indicated substantial variability (*p* < 0.001, *I²* = 89.6%, 95% PI [-1.55, 0.42]). The results of the meta-analysis showed that compared with the control group, JLD significantly reduced TC (MD = -0.57, 95% CI: -0.87 to -0.27; *p* < 0.001). The corresponding forest plot is presented in **Figure 10**.

Subgroup analyses suggested greater TC reductions in T2DM patients (MD = -0.75, 95% CI: -1.00 to -0.50; *p* for interaction = 0.014, *P*_Holm = 0.057, R² = 71.6%). Patients with baseline glucose ≥10.0 mmol/L experienced larger benefits (MD = -0.92, 95% CI: -1.17 to -0.67); however, the subgroup difference was not statistically significant (*p* = 0.037, *P*_Holm = 0.112, R² = 75.8%). No significant subgroup effects were found for trial duration (*p* = 0.301, *P*_Holm = 0.301, R² = 11.8%) or age (*p* = 0.148, *P*_Holm = 0.296, R² = 11.4%). Detailed results are provided in **Supplementary Table 7**.

#### 3.4.8 Effect of JLD on TG

**Figure 11:**
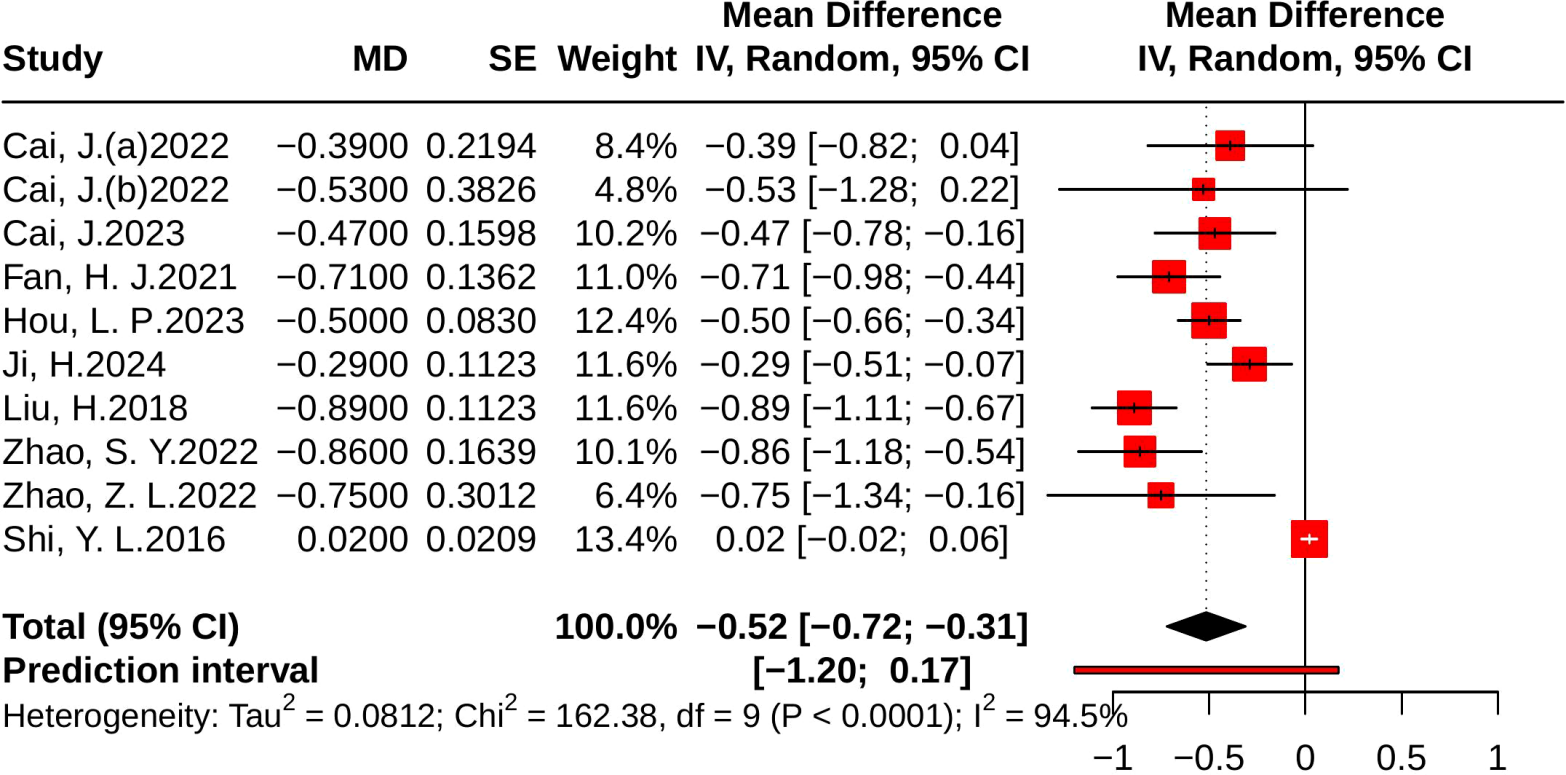
Forest plots of the effect of JLD on TG. Each study’s estimate is plotted as a square scaled to weight, with 95% CIs shown as horizontal lines. The pooled MD from the random-effects model is displayed as a diamond, and the line at 0 indicates no difference.

Ten studies evaluated TG, including 849 participants in the control group and 845 in the treatment group. Heterogeneity analysis revealed substantial heterogeneity (*p* < 0.001, *I²* = 94.5%, 95% PI [-1.20, 0.17]). The meta-analysis showed that, compared with the control group, JLD significantly reduced TG levels (MD = -0.52, 95% CI: -0.72 to -0.31; *p* < 0.001), as shown in **Figure 11**.

Subgroup analyses indicated greater TG reductions in T2DM patients (MD = -0.65, 95% CI: -0.80 to -0.50; *p* for interaction = 0.003, *P*_Holm = 0.013, R² = 69.7%). Patients with baseline glucose ≥10.0 mmol/L experienced larger benefits (MD = -0.87, 95% CI: -1.08 to -0.67), and subgroup differences remained statistically significant (*p* = 0.004, *P*_Holm = 0.013, R² = 78.5%). No significant subgroup effects were found for trial duration (*p* = 0.099, *P*_Holm = 0.198, R² = 24.5%) or age (*p* = 0.948, *P*_Holm = 0.948, R² = 0%), as illustrated in **Supplementary Table 8**.

#### 3.4.9 Adverse events

**Figure 12:**
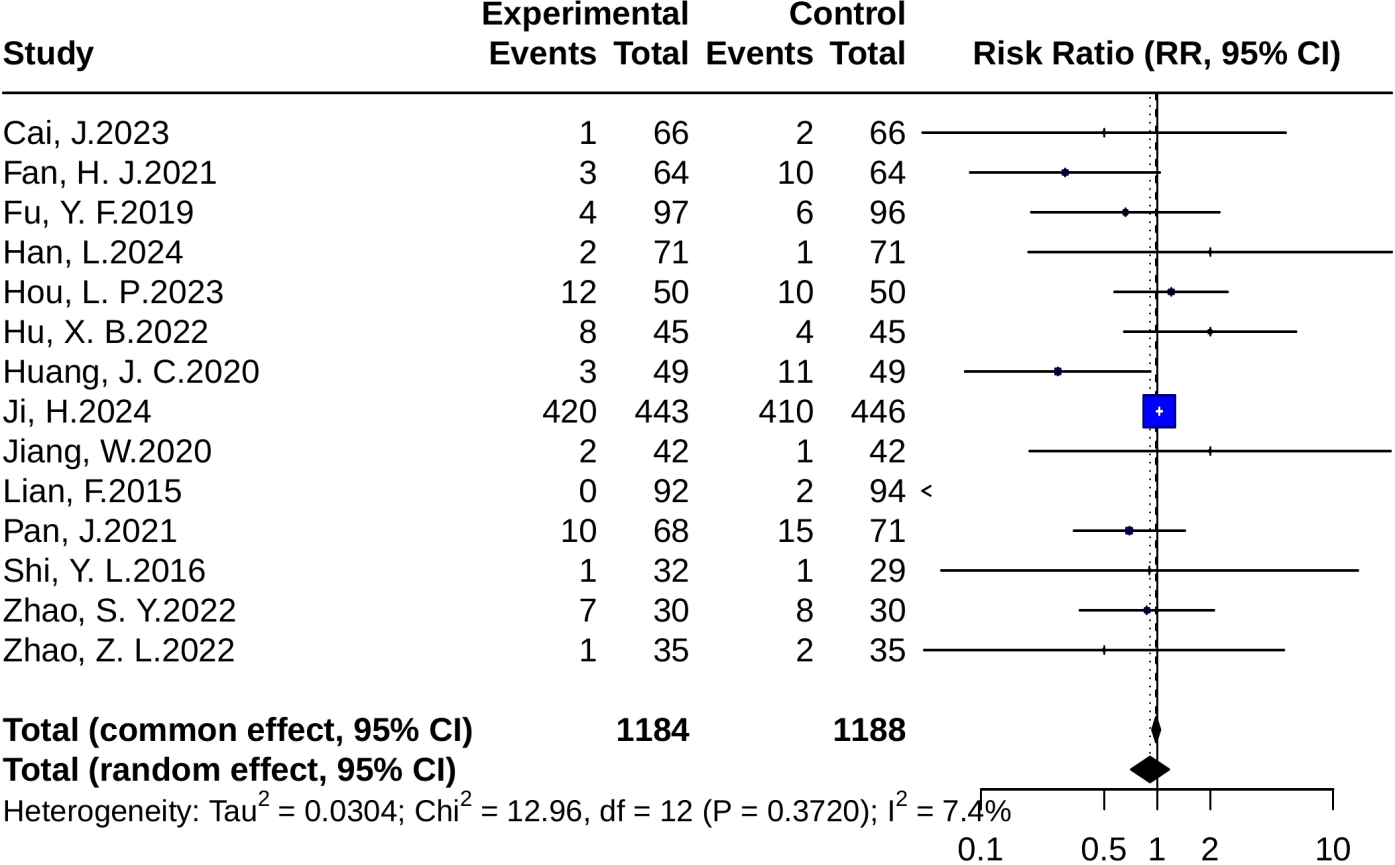
Forest plots of adverse event risk (RR). Squares represent risk ratios (RRs) from individual studies, weighted by study precision. Horizontal bars show 95% CIs. The pooled estimate, obtained with a random-effects model, is represented by a diamond. The vertical line at 1 denotes the null value for risk comparison.

In the safety analysis, a total of 14 randomized controlled trials were included. The pooled results from the random-effects model showed no significant difference in the incidence of adverse events between the intervention and control groups (RR = 0.91, 95% CI: 0.71–1.17; *p* = 0.467). The heterogeneity test indicated *p* = 0.37 and *I²* = 7.4%, suggesting low between-study heterogeneity, as illustrated in **Figure 12**.

#### 3.4.10 Meta-regression

The results of this meta-analysis indicated that most outcomes exhibited substantial heterogeneity. For outcomes with more than 10 included studies, meta-regression was conducted. The covariates (trial duration, age, baseline FBG, background treatment, and status) explained a large proportion of the heterogeneity in FBG (R² = 81.02%) and HbA1c (R² = 62.98%), with trial duration (<12 weeks) emerging as a significant moderator for both outcomes. For 2hPG, the model explained nearly half of the heterogeneity (R² = 49.93%), although the overall test of moderators was not statistically significant (*p* = 0.079). For TG, the included moderators accounted for most of the variability (R² = 69.18%), but no individual covariate reached statistical significance. By contrast, the meta-regression model for HOMA-IR yielded a negative R² (-35.73%), suggesting that the selected covariates did not explain the between-study variability and may even have worsened the model fit. Detailed results are presented in **Supplementary Tables 9–13**.

#### 3.4.11 Sensitivity analyses

To assess the robustness of our findings, several sensitivity analyses were performed. Leave-one-out analysis showed that no single study had a decisive influence on the pooled effect, with results remaining stable (**Supplementary Figures 1–8**). Excluding high-risk-of-bias studies yielded results consistent with the main analysis (**Supplementary Table 14**). Similarly, removing trials without standardized Western medicine background interventions did not materially alter the pooled estimates (**Supplementary Table 15**). Influence diagnostics identified a few studies with some impact on model fit, but exclusion of these studies did not substantially change the results (**Supplementary Figures 9–16**). For sparse binary outcomes, alternative continuity correction methods were applied to account for zero-event studies, and the results remained consistent (**Supplementary Figure 17**). Overall, these analyses indicate that our conclusions are robust and not driven by any single study, specific subgroup of studies, or methodological choice.

### 3.5 Publication bias

**Figure 13:**
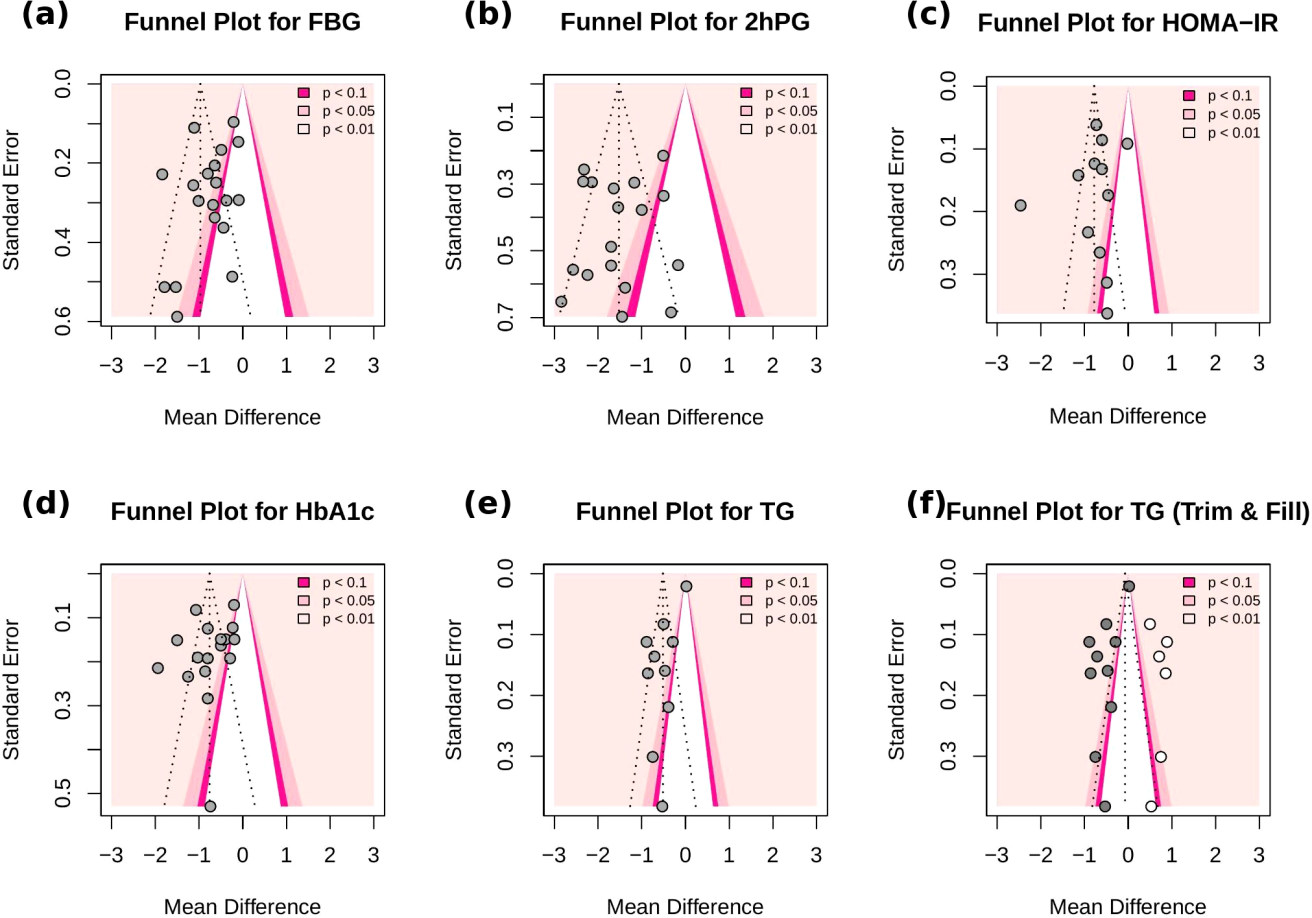
Funnel plots for **(a)** FBG, **(b)** 2h-PG, **(c)** HOMA-IR, **(d)** HbA1c, **(e)** TG **(f)** TG (Trim-and-fill). Each dot represents a study included in the meta-analysis. The vertical line denotes the pooled effect size, and the diagonal dashed lines represent the expected 95% confidence intervals around the effect. Grey dots correspond to the original studies, while white dots indicate imputed studies added by the trim-and-fill method.

Publication bias was assessed using funnel plots and Egger’s test. The funnel plots for FBG, 2hPG, HOMA-IR, and HbA1c appeared largely symmetrical, with Egger’s test yielding non-significant results (*p* = 0.27, *p* = 0.64, *p* = 0.45, and *p* = 0.23, respectively), suggesting no evidence of substantial publication bias. For the TG outcome, the funnel plot showed some asymmetry, and Egger’s test (*p* < 0.05) suggested potential publication bias. Before trim-and-fill correction, the pooled effect was significant (MD = -0.52, 95% CI -0.72 to -0.31, *p* < 0.001). After applying the trim-and-fill method, the adjusted pooled effect size was attenuated and became non-significant (MD = -0.07, 95% CI -0.40 to 0.25, *p* = 0.661), suggesting that the initial significance may have been influenced by publication bias (**Figure 13**).

### 3.6 Quality of evidence

The GRADE assessment indicated that, due to risk of bias and inconsistency across the included studies, the overall certainty of evidence was rated as low for most outcomes, including FPG, 2h-PG, HDL-C, LDL-C, HOMA-IR, HbA1c, and TC. In addition, the certainty of evidence for TG was downgraded further to very low because of the combined influence of risk of bias, inconsistency, and potential publication bias. Detailed explanations of the ratings and reasons for downgrading are summarized in **Table 2**.

**Table 2:** GRADE evidence profile for primary outcomes.

Outcomes	Certainty assessment						No. of patients	Effect sizes	Certainty
	No. of trials	Risk of bias	Inconsistency	Indirectness	Imprecision	Publication bias	Intervention/Control	Summary effect estimates (95% CI)	
FPG	21	Serious	Serious	NO Serious	NO Serious	NO Serious	1469/1467	MD = –0.97, 95% CI: –1.40 to –0.53	⊕⊕⊝⊝ Low
2h-PG	19	Serious	Serious	NO Serious	NO Serious	NO Serious	1390/1391	MD = –1.52 95% CI: –1.89 to –1.16	⊕⊕⊝⊝ Low
HDL-C	8	Serious	Serious	NO Serious	NO Serious	Undetected	723/719	MD = 0.22, 95% CI: 0.12 to 0.32	⊕⊕⊝⊝ Low
LDL-C	9	Serious	Serious	NO Serious	NO Serious	Undetected	783/779	MD = –0.69, 95% CI: –1.05 to –0.33	⊕⊕⊝⊝ Low
HOMA-IR	12	Serious	Serious	NO Serious	NO Serious	NO Serious	989/983	MD = –0.78, 95% CI: –1.12 to –0.44	⊕⊕⊝⊝ Low
Hba1c	18	Serious	Serious	NO Serious	NO Serious	NO Serious	1340/1343	MD = –0.76, 95% CI: –1.00 to –0.52	⊕⊕⊝⊝ Low
TC	9	Serious	Serious	NO Serious	NO Serious	Undetected	814/810	MD = –0.57, 95% CI: –0.87 to –0.27	⊕⊕⊝⊝ Low
TG	10	Serious	Serious	NO Serious	NO Serious	Serious	849/845	MD = −0.52, 95% CI: −0.72 to −0.31	⊕⊝⊝⊝ Very Low
Adverse events	14	Serious	NO Serious	NO Serious	NO Serious	No serious	1408/1412	RR = 0.91, 95% CI: 0.71–1.17	⊕⊕⊕⊝Moderate

GRADE, Grading of Recommendations Assessment, Development, and Evaluation:

*Risk of bias*: serious, study with unclear risk of bias.

*Inconsistency*: Serious, *I^2^*>50%.

*Indirectness*: no indirectness of evidence was found in any study.

*Imprecision (based on sample size)*: Serious, n < 500 participants.

*Publication bias*: The Egger’s test indicated potential publication bias.

## 4 Discussion

### 4.1 Main finding

This study investigated the effects of JLD on metabolic outcomes in patients with PD and T2DM. The results indicated that JLD, in addition to standard therapy, significantly lowered FBG, 2h-PG, and HbA1c relative to the control group, highlighting meaningful improvements in glycemic regulation. JLD was also effective in reducing insulin resistance, as reflected by favorable changes in HOMA-IR, and exerted positive effects on lipid profiles by decreasing TC, TG, and LDL-C, while simultaneously increasing HDL-C.

The subgroup analyses provide important insights into the populations most likely to benefit from JLD. Greater improvements were generally observed in shorter trials, suggesting an early therapeutic effect that may diminish or plateau with longer treatment, underscoring the need to evaluate strategies for sustaining long-term efficacy. Baseline glycemic status emerged as a key effect modifier. Patients with fasting glucose ≥10 mmol/L achieved the largest reductions in both fasting and postprandial glucose, as well as more favorable lipid changes, indicating that JLD may be particularly effective in metabolically uncontrolled populations. Compared with PD, patients with T2DM showed more pronounced improvements in HbA1c, HOMA-IR, and lipid profiles. This is likely attributable to their higher baseline glucose levels and more severe β-cell dysfunction, which make them more responsive to pharmacologic or nutritional interventions targeting glucose homeostasis and insulin resistance (46, 47). Nevertheless, even modest reductions in fasting or postprandial glucose among PD participants are clinically meaningful, as they may delay progression to overt diabetes and reduce the risk of long-term complications (48). Overall, these findings suggest that JLD provides clear therapeutic value for T2DM patients with poor metabolic control, while its role as an early intervention in PD remains promising but requires confirmation in larger, long-term studies.

Meta-regression analyses identified trial duration and baseline FBG as significant contributors to between-study heterogeneity. Shorter intervention durations and higher baseline FBG levels were associated with greater reductions in FBG and HbA1c, suggesting a stronger early-phase effect of JLD, which may plateau over time. Background therapy, defined as the use of concomitant glucose-lowering medications, was also included as a moderator. Although it did not reach statistical significance, the direction of effect was consistent: studies without background medications tended to report larger glycemic improvements (e.g., FBG: *β* = 0.96, 95% CI −0.10 to 2.01, *p* = 0.076). This trend may be partially explained by a ceiling effect, whereby patients already receiving hypoglycemic drugs have a limited capacity for further glucose reduction. In contrast, in trials in which JLD was administered as monotherapy, a broader therapeutic window may allow its effects to be more fully observed. Background therapy was not a statistically significant independent predictor. However, its inclusion in the FBG model (R² = 81%) suggests a potential interaction with other moderators. This model explained the largest proportion of heterogeneity, indicating its relevance to treatment response. These findings underscore the need to standardize and report background treatments clearly in clinical trials, as variability in co-interventions may otherwise obscure or exaggerate the observed efficacy of the intervention.

### 4.2 Comparison of similar studies

Two prior meta-analyses have summarized the clinical efficacy of JLD in patients with T2DM, yet their conclusions were not fully consistent. Lian et al. synthesized 15 RCTs involving 1,810 participants and reported significant reductions in HbA1c, FPG, and 2h-PG (19). In contrast, Zhao et al. screened 22 RCTs but, due to methodological restrictions, included only three low–risk–of–bias studies (441 participants) in quantitative synthesis (18). Their analysis showed only a marginal decrease in HbA1c (MD −0.283%, 95% CI −0.561 to −0.004; p = 0.046) and no significant changes in FPG or 2h-PG. Thus, while Lian et al. suggested clear glycemic benefits, Zhao et al. reported more conservative estimates based on stricter inclusion criteria. Building upon this foundation, the present study expands the evidence base both quantitatively and conceptually. We extended the search to July 2025 and identified 20 randomized controlled trials including 2,993 participants. The updated evidence base also incorporated several recently published high-quality studies. In addition to patients with established T2DM, we included those with prediabetes, allowing for a broader assessment across the spectrum of dysglycemia. Beyond the three glycemic endpoints emphasized previously, our analysis systematically synthesized outcomes related to HOMA-IR, lipid metabolism (TC, TG, LDL-C, HDL-C), and safety, thereby constructing a more comprehensive profile of JLD’s metabolic effects. Methodologically, this study performed comprehensive sensitivity analyses to assess the robustness of pooled estimates across different statistical models and study quality levels. Furthermore, this study also introduces subgroup analyses and multivariable random-effects meta-regression to explore how participant characteristics and intervention parameters influence therapeutic outcomes—an approach not undertaken in earlier reviews. These analyses revealed that individuals with higher baseline glycemia or established T2DM tended to achieve greater short-term glycemic improvements, while those with prediabetes showed potential preventive benefits. Moreover, trials with shorter treatment duration or lighter background therapy demonstrated larger effect sizes.

Collectively, our findings refine and extend the conclusions of earlier reviews. Compared with Zhao et al., who reported only modest glycemic benefits and limited interpretability due to the small number of studies, our results confirm consistent improvements in HbA1c, FPG, and 2h-PG and further demonstrate significant enhancements in insulin sensitivity and lipid regulation. This multidimensional evidence framework provides an updated and clinically relevant understanding of JLD’s efficacy and safety across different stages of metabolic dysregulation. By quantitatively analyzing how participant characteristics and treatment parameters shape these outcomes, our study offers practical guidance for optimizing therapeutic strategies and supports more targeted, effective, and individualized TCM interventions that benefit diverse patient populations.

### 4.3 Mechanisms of JLD

JLD is derived from a traditional Chinese medicine (TCM) formulation that adheres to the therapeutic principles of “tonifying Qi, nourishing Yin, strengthening the spleen, and promoting fluid metabolism.” It comprises multiple herbal components with demonstrated hypoglycemic properties. Evidence from animal experiments and preclinical studies indicates that JLD regulates glucose and lipid metabolism through multiple mechanisms. Specifically, JLD has been shown to stimulate brown adipose tissue (BAT) thermogenesis by enhancing mitochondrial biogenesis and promoting fatty acid oxidation (49). It also improves insulin signaling and reduces JNK and p38 MAPK phosphorylation, thereby counteracting high-fat-diet–induced hyperglycemia, hyperinsulinemia, and insulin resistance, while also exerting antioxidant activity (50). Furthermore, JLD activates AMPK to protect β-cells and stimulates the PPARα/ABCA1 pathway, thereby improving glucose–lipid metabolism, reducing hepatic lipid deposition, and alleviating insulin resistance (51). In addition, JLD may slow β-cell aging and suppress the release of SASP-related cytokines, thereby improving insulin secretion and correcting metabolic dysfunction in PD (52). *In vitro* experiments have further demonstrated that JLD enhances insulin secretion, promotes *β*-cell proliferation, and inhibits apoptosis under high-glucose conditions. Mechanistically, JLD increases the expression of the anti-apoptotic protein Bcl-2, decreases pro-apoptotic proteins such as Caspase-3 and Bax, and prevents excessive phosphorylation of Smad2/3 within the insulin signaling pathway (53). Collectively, these findings suggest that JLD exerts antidiabetic effects through dual mechanisms: improving insulin resistance and preserving islet function. Additionally, network pharmacology analyses have identified several active compounds in JLD, including quercetin and luteolin, which target key signaling pathways such as PI3K-AKT, PTGS2, and AGE–RAGE. These pathways are critically involved in regulating signal transduction, apoptosis, gene expression, and inflammatory responses (54).

### 4.4 Limitations and future research

This review has several limitations that should temper interpretation. First, between-study heterogeneity was substantial. Meta-regression suggested that intervention duration and baseline FBG explained a meaningful proportion of the variability in glycemic outcomes; background therapy was also modeled as a moderator. Although this term did not consistently reach statistical significance, its direction was concordant with the overall pattern of findings, indicating that differences in background care likely contributed to the observed dispersion. Second, methodological quality varied across studies. This variation was especially evident in trials conducted in China before 2018. Such differences may have influenced the pooled estimates. The adoption of CONSORT-aligned practices has historically been incomplete. Prospective registration and public availability of protocols or statistical analysis plans (SAPs) were often limited. Many studies used small, single-center designs with constrained monitoring. In addition, practical barriers to robust allocation concealment and multi-level blinding likely introduced domain-specific biases. Inadequate sequence generation or concealment can lead to selection bias, which often inflates effect estimates. Limited blinding of participants, personnel, or outcome assessors may introduce performance or detection bias. The direction of such bias is uncertain and may depend on co-interventions and how outcomes are measured. Additional concerns include the inconsistent handling of missing data and occasional reliance on per-protocol rather than intention-to-treat analyses, both of which can shift estimates toward statistical significance. To empirically address these risks within this methodological context, we prespecified and conducted a sensitivity analysis excluding trials rated as high risk of bias according to RoB 2.0. After exclusion, the pooled effects for primary outcomes remained directionally consistent and statistically significant, with only modest attenuation, suggesting that our conclusions are not materially driven by high-risk designs. Nonetheless, this check has inherent constraints—exclusions reduce power, incomplete reporting of concomitant therapy leaves room for residual confounding, and earlier small studies may differ systematically from later trials—so the magnitude of the effect should be interpreted with caution. Third, evidence in PD remains limited. Although two relatively large RCTs were included, the overall number of PD trials was small, limiting the precision and generalizability of subgroup estimates. Fourth, all included studies used the same labeled dose of JLD. This uniformity prevents any assessment of dose–response relationships. It also makes comparisons across different dosing regimens impossible. As a result, the optimal dose and the exposure–response relationship cannot be determined based on the current evidence. Finally, all included studies were conducted in China, which may restrict external validity across other health systems, ethnicities, and care pathways.

Future research should address the following priorities to strengthen the evidence base. Research should prioritize multicenter, adequately powered RCTs with prospective registration and publicly available protocols/SAPs; rigorous randomization with allocation concealment (e.g., centralized systems or opaque sequential allocation) and blinding (or blinded outcome adjudication when double blinding is infeasible); intention-to-treat analyses with transparent handling of missing data; and harmonized outcome measurements, including core outcome sets, standardized assay methods and units, and prespecified time points. Greater population diversity and longer follow-up periods are needed to evaluate clinical endpoints (e.g., cardiovascular outcomes) and to determine the optimal treatment duration and combination strategies with standard care. In addition, dose-finding and dose-ranging trials (including multi-arm designs) and modeling-based exposure–response analyses are warranted to identify the minimal effective and optimal dosing of JLD and to test whether benefits differ across dosing strategies. Finally, mechanistic and pharmacokinetic/pharmacodynamic studies are warranted to clarify the underlying biological pathways and to strengthen causal inference regarding JLD’s glycemic and lipid effects.

## 5 Conclusion

This study indicates that JLD has potential efficacy in improving glycemic control and insulin resistance in individuals with PD and T2DM. Additionally, JLD exerts favorable effects on lipid metabolism by lowering TC, TG, and LDL-C, while simultaneously increasing HDL-C levels, with lipid-modulating effects being more pronounced in T2DM patients. Subgroup analyses further revealed that greater benefits were observed in shorter-duration trials and in patients with higher baseline fasting glucose, suggesting that JLD may be particularly effective in metabolically uncontrolled populations. Overall, the findings suggest that JLD may serve as an effective adjuvant therapy for the management of T2DM and PD. Nevertheless, several limitations should be acknowledged, including considerable heterogeneity across included studies and relatively small sample sizes in some subgroups, which may reduce the statistical robustness of the findings. Future research should prioritize rigorously designed, large-scale, multicenter RCTs to further substantiate the efficacy and safety of JLD across diverse populations and clarify its clinical applicability.

## Data Availability

The original contributions presented in the study are included in the article/**Supplementary Material**. Further inquiries can be directed to the corresponding authors.
