# Supplementary material for: The impact of Jinlida on blood glucose control and insulin resistance in patients with prediabetes and type 2 diabetes: a systematic review and meta-analysis of randomized controlled trials
Source: Front Endocrinol (Lausanne). 2025 Nov 17;16:1689640. doi: 10.3389/fendo.2025.1689640 (PMC12666531; doi:10.3389/fendo.2025.1689640)
Supplement: Supplementary file 1 [file DataSheet1.docx]

search strategy

**1、Pubmed：**

**(Jinlida) AND ((("Prediabetic State"[Mesh]) OR ("Diabetes Mellitus, Type 2"[Mesh])) OR ((((((type 2 diabetes) OR (T2D)) OR (pre-diabetes)) OR (pre diabetes)) OR (diabetes)) OR (T2DM)))**

**2、Embase**

#4 AND #5

#5 jinlida

#4 #1 OR #2 OR #3

#3 'non insulin dependent diabetes mellitus'/exp

#2 'impaired glucose tolerance'/exp

#1 'type 2 diabetes'/exp OR 'type 2 diabetes' OR (type AND ('2'/exp OR 2) AND ('diabetes'/exp OR diabetes)) OR prediabetes OR 'pre diabetes' OR t2d OR diabetes

**3、Web of science**

(((((ALL=(diabetes)) OR ALL=(type 2 diabetes)) OR ALL=(T2D)) OR ALL=(pre-diabetes)) OR ALL=(pre diabetes)) AND ALL=(Jinlida)

**4、Cochrane:**

#1 (diabetes):ti,ab,kw OR (type 2 diabetes):ti,ab,kw OR (T2D):ti,ab,kw OR (pre-diabetes):ti,ab,kw OR (prediabetes):ti,ab,kw

#2 Jinlida

#3 #1 AND #2

**5、Scopus**

( TITLE-ABS-KEY ( jinlida ) ) AND ( ( TITLE-ABS-KEY ( diabetes ) OR TITLE-ABS-KEY ( type 2 diabetes ) OR TITLE-ABS-KEY ( t2d ) OR TITLE-ABS-KEY ( pre-diabetes ) OR TITLE-ABS-KEY ( pre AND diabetes ) ) )

**6、CNKI：**

( TKA % '津力达' ) AND ( TKA % '糖尿病' OR TKA % '糖尿病前期' OR TKA % '糖前期' OR TKA % '血糖')

**7、Wangfang：**

(( 摘要:(糖尿病) or 摘要:(糖前期) or 摘要:(糖尿病前期) or 摘要:(血糖))and 摘要:(津力达))

**8、VIP：**

(M=糖尿病 OR M= 糖前期 OR M= 糖尿病前期 OR M= 血糖) AND M= 津力达

**9、China Biology Medicine Database (CBM)：**

("糖尿病"[常用字段:智能] OR "糖尿病前期"[常用字段:智能] OR "糖前期"[常用字段:智能] OR "血糖"[常用字段:智能]) AND ("津力达"[常用字段:智能])
