# Supplementary material for: The impact of Jinlida on blood glucose control and insulin resistance in patients with prediabetes and type 2 diabetes: a systematic review and meta-analysis of randomized controlled trials
Source: Front Endocrinol (Lausanne). 2025 Nov 17;16:1689640. doi: 10.3389/fendo.2025.1689640 (PMC12666531; doi:10.3389/fendo.2025.1689640)
Supplement: Supplementary file 2 [file DataSheet2.docx]

Supplementary Material

# Supplementary Data

Supplementary Material should be uploaded separately on submission. Please include any supplementary data, figures and/or tables.

Supplementary material is not typeset so please ensure that all information is clearly presented, the appropriate caption is included in the file and not in the manuscript, and that the style conforms to the rest of the article.

# Supplementary Figures and Tables

# 2.1 Supplementary Tables

**Supplementary Table 1. Subgroup analysis of the effect of JLD on changes in FBG**

| **Subgroup** | **Group** | **No. of trials** | **No of participants** | **Effect size (MD [95% CI])** | **P** | **I² (%)** | **τ²** | ***P* for Interaction** | **R² (%)** | ***P_Holm*** |
| --- | --- | --- | --- | --- | --- | --- | --- | --- | --- | --- |
| **Trial duration (weeks)** | <12 | 4 | 230/230 | -2.23 [-3.71, -0.74] | 0.0033 | 100 | 1.48 | 0.0012 | 45.63 | 0.00371 |
|  | ≥12 | 16 | 1266/1267 | -0.58 [-0.78, -0.39] | <0.0001 | 60 | 0.287 |  |  |  |
| **Health status** | PD | 2 | 474/472 | -0.18 [-0.33, -0.02] | 0.028 | 0 | 0 | 0.201 | 4.1 | 0.401 |
|  | T2DM | 18 | 1022/1025 | -1.07 [-1.53, -0.60] | <0.0001 | 90 | 0.953 |  |  |  |
| **Baseline FBG level (mmol/L)** | <7.0 | 2 | 474/472 | -0.18 [-0.33, -0.02] | 0.028 | 0 | 0 | 0.0005 | 62.61 | 0.00204 |
|  | ≥10.0 | 3 | 150/150 | -2.62 [-4.48, -0.76] | 0.0057 | 90 | 1.574 |  |  |  |
|  | 7.0–9.9 | 15 | 872/875 | -0.79 [-1.02, -0.55] | <0.0001 | 70 | 0.373 |  |  |  |
| **Age** | <45 | 1 | 97/96 | -0.64 [-1.04, -0.24] | 0.0019 | NA | NA | 0.366 | 0.51 | 0.401 |
|  | 45–59 | 14 | 1131/1133 | -0.77 [-1.08, -0.47] | <0.0001 | 90 | 0.507 |  |  |  |
|  | 60–74 | 5 | 268/268 | -1.52 [-3.00, -0.03] | 0.045 | 100 | 1.666 |  |  |  |

P_Holm, Holm-adjusted P value for multiple comparisons.

**Supplementary Table 2.** Subgroup analysis of the effect of JLD on changes in 2hPG

| **Subgroup** | **Group** | **No. of trials** | **No of participants** | **Effect size (MD [95% CI])** | **P** | **I² (%)** | **τ²** | **P for Interaction** | **R² (%)** | ***P_Holm*** |
| --- | --- | --- | --- | --- | --- | --- | --- | --- | --- | --- |
| **Trial duration (weeks)** | <12 | 4 | 230/230 | -2.22 [-2.52, -1.92] | <0.001 | 0 | 0 | 0.025 | 44.53 | 0.101 |
|  | ≥12 | 14 | 1160 1161 | -1.30 [-1.69, -0.90] | <0.001 | 70 | 0.593 |  |  |  |
| **Health status** | PD | 2 | 474/472 | -1.00 [-2.15, 0.15] | 0.088 | 80 | 0.732 | 0.279 | 7.28 | 0.557 |
|  | T2DM | 16 | 1022/1025 | -1.60 [-1.97, -1.22] | <0.001 | 70 | 0.616 |  |  |  |
| **Baseline FBG level (mmol/L)** | <7.0 | 2 | 474/472 | -1.00 [-2.15, 0.15] | 0.088 | 80 | 0.732 | 0.173 | 19.34 | 0.519 |
|  | ≥10.0 | 3 | 150/150 | -2.24 [-2.69, -1.79] | <0.001 | 0 | 0 |  |  |  |
|  | 7.0–9.9 | 13 | 766/769 | -1.46 [-1.87, -1.04] | <0.001 | 70 | 0.618 |  |  |  |
| **Age** | <45 | 1 | 97/96 | -1.17 [-1.75, -0.59] | <0.001 | NA | NA | 0.412 | 0 | 0.557 |
|  | 45–59 | 12 | 1025/1027 | -1.40 [-1.86, -0.94] | <0.001 | 80 | 0.687 |  |  |  |
|  | 60–74 | 5 | 268/268 | -1.94 [-2.62, -1.27] | <0.001 | 60 | 0.582 |  |  |  |

**Supplementary Table 3.** Subgroup analysis of the effect of JLD on changes in HbA1c

| **Subgroup** | **Group** | **No. of trials** | **No of participants** | **Effect size (MD [95% CI])** | **P** | **I² (%)** | **τ²** | **P for Interaction** | **R² (%)** | ***P_Holm*** |
| --- | --- | --- | --- | --- | --- | --- | --- | --- | --- | --- |
| **Trial duration (weeks)** | <12 | 4 | 219/219 | -1.21 [-1.68, -0.75] | <0.001 | 80 | 0.435 | 0.029 | 27 | 0.116 |
|  | ≥12 | 13 | 1121/1124 | -0.61 [-0.85, -0.38] | <0.001 | 90 | 0.384 |  |  |  |
| **Health status** | PD | 2 | 474/472 | -0.32 [-0.61, -0.03] | 0.029 | 70 | 0.177 | 0.187 | 6.2 | 0.562 |
|  | T2DM | 15 | 866/871 | -0.82 [-1.08, -0.56] | <0.001 | 90 | 0.462 |  |  |  |
| **Baseline FBG level (mmol/L)** | <7.0 | 2 | 474/472 | -0.32 [-0.61, -0.03] | 0.029 | 70 | 0.177 | 0.433 | 0 | 0.866 |
|  | ≥10.0 | 2 | 90/90 | -0.80 [-1.11, -0.49] | <0.001 | 0 | 0 |  |  |  |
|  | 7.0–9.9 | 13 | 776/781 | -0.82 [-1.12, -0.53] | <0.001 | 90 | 0.501 |  |  |  |
| **Age** | <45 | 1 | 97/96 | -0.86 [-1.30, -0.42] | <0.001 | NA | NA | 0.796 | 0 | 0.866 |
|  | 45–59 | 12 | 1035/1039 | -0.71 [-1.01, -0.41] | <0.001 | 90 | 0.497 |  |  |  |
|  | 60–74 | 4 | 208/208 | -0.91 [-1.39, -0.43] | <0.001 | 90 | 0.431 |  |  |  |

**Supplementary Table 4.** Subgroup analysis of the effect of JLD on changes in HOMA-IR

| **Subgroup** | **Group** | **No. of trials** | **No of participants** | **Effect size (MD [95% CI])** | **P** | **I² (%)** | **τ²** | **P for Interaction** | **R² (%)** | ***P_Holm*** |
| --- | --- | --- | --- | --- | --- | --- | --- | --- | --- | --- |
| **Trial duration (weeks)** | <12 | 1 | 64/64 | -0.73 [-0.85, -0.61] | <0.001 | NA | NA | 0.934 | 0 | 1.000 |
|  | ≥12 | 11 | 925/919 | -0.78 [-1.16, -0.41] | <0.001 | 90 | 0.604 |  |  |  |
| **Health status** | PD | 2 | 474/472 | -0.49 [-0.95, -0.02] | 0.04 | 0 | 0 | 0.522 | 0 | 1.000 |
|  | T2DM | 10 | 515/511 | -0.83 [-1.22, -0.43] | <0.001 | 90 | 0.615 |  |  |  |
| **Baseline FBG level (mmol/L)** | <7.0 | 2 | 474/472 | -0.49 [-0.95, -0.02] | 0.04 | 0 | 0 | 0.763 | 0 | 1.000 |
|  | ≥10.0 | 1 | 30/30 | -0.60 [-0.86, -0.34] | <0.001 | NA | NA |  |  |  |
|  | 7.0–9.9 | 9 | 485/481 | -0.85 [-1.29, -0.42] | <0.001 | 90 | 0.65 |  |  |  |
| **Age** | 45–59 | 9 | 831/829 | -0.65 [-0.89, -0.41] | <0.001 | 90 | 0.311 | 0.215 | 5.28 | 0.862 |
|  | 60–74 | 3 | 158/154 | -1.17 [-2.42, 0.09] | 0.069 | 100 | 1.099 |  |  |  |

**Supplementary Table 5.** Subgroup analysis of the effect of JLD on changes in HDL-C

| **Subgroup** | **Group** | **No. of trials** | **No of participants** | **Effect size (MD [95% CI])** | **P** | **I² (%)** | **τ²** | **P for Interaction** | **R² (%)** | ***P_Holm*** |
| --- | --- | --- | --- | --- | --- | --- | --- | --- | --- | --- |
| **Trial duration (weeks)** | <12 | 1 | 64/64 | 0.32 [0.09, 0.55] | 0.007 | NA | NA | 0.564 | 0 | 1.000 |
|  | ≥12 | 7 | 659/655 | 0.21 [0.10, 0.32] | <0.001 | 90 | 0.134 |  |  |  |
| **Health status** | PD | 2 | 474/472 | 0.01 [-0.02, 0.05] | 0.435 | 0 | 0 | 0.003 | 84.2 | 0.012 |
|  | T2DM | 6 | 249/247 | 0.28 [0.21, 0.35] | <0.001 | 50 | 0.061 |  |  |  |
| **Baseline FBG level (mmol/L)** | <7.0 | 2 | 474/472 | 0.01 [-0.02, 0.05] | 0.435 | 0 | 0 | 0.005 | 89.1 | 0.015 |
|  | ≥10.0 | 1 | 30/30 | 0.39 [0.26, 0.52] | <0.001 | NA | NA |  |  |  |
|  | 7.0–9.9 | 5 | 219/217 | 0.26 [0.19, 0.33] | <0.001 | 40 | 0.052 |  |  |  |
| **Age** | 45–59 | 7 | 673/669 | 0.20 [0.09, 0.32] | <0.001 | 90 | 0.135 | 0.528 | 0 | 1.000 |
|  | 60–74 | 1 | 50/50 | 0.31 [0.17, 0.45] | <0.001 | NA | NA |  |  |  |

**Supplementary Table 6.** Subgroup analysis of the effect of JLD on changes in LDL-C

| **Subgroup** | **Group** | **No. of trials** | **No of participants** | **Effect size (MD [95% CI])** | **P** | **I² (%)** | **τ²** | **P for Interaction** | **R² (%)** | ***P_Holm*** |
| --- | --- | --- | --- | --- | --- | --- | --- | --- | --- | --- |
| **Trial duration (weeks)** | <12 | 2 | 124/124 | -0.99 [-1.14, -0.83] | <0.001 | 0 | 0 | 0.442 | 0 | 0.442 |
|  | ≥12 | 7 | 659/655 | -0.61 [-1.06, -0.16] | 0.008 | 90 | 0.58 |  |  |  |
| **Health status** | PD | 2 | 474/472 | -0.03 [-0.12, 0.07] | 0.57 | 10 | 0.03 | 0.033 | 52.4 | 0.133 |
|  | T2DM | 7 | 309/307 | -0.89 [-1.22, -0.56] | <0.001 | 80 | 0.407 |  |  |  |
| **Baseline FBG level (mmol/L)** | <7.0 | 2 | 474/472 | -0.03 [-0.12, 0.07] | 0.57 | 10 | 0.03 | 0.128 | 41.4 | 0.385 |
|  | ≥10.0 | 2 | 219/217 | -0.84 [-1.08, -0.59] | <0.001 | 0 | 0 |  |  |  |
|  | 7.0–9.9 | 5 | 90/90 | -0.92 [-1.41, -0.43] | <0.001 | 90 | 0.519 |  |  |  |
| **Age** | 45–59 | 8 | 733/729 | -0.60 [-0.95, -0.26] | <0.001 | 100 | 0.478 | 0.143 | 16.1 | 0.385 |
|  | 60–74 | 1 | 50/50 | -1.58 [-2.20, -0.96] | <0.001 | NA | NA |  |  |  |

**Supplementary Table 7** Subgroup analysis of the effect of JLD on changes in TC

| **Subgroup** | **Group** | **No. of trials** | **No of participants** | **Effect size (MD [95% CI])** | **P** | **I² (%)** | **τ²** | **P for Interaction** | **R² (%)** | ***P_Holm*** |
| --- | --- | --- | --- | --- | --- | --- | --- | --- | --- | --- |
| **Trial duration (weeks)** | <12 | 2 | 124/124 | -0.88 [-1.14, -0.62] | <0.001 | 0 | 0 | 0.301 | 11.8 | 0.301 |
|  | ≥12 | 7 | 690/686 | -0.48 [-0.83, -0.13] | 0.007 | 90 | 0.416 |  |  |  |
| **Health status** | PD | 2 | 474/472 | -0.03 [-0.24, 0.18] | 0.746 | 60 | 0.122 | 0.014 | 71.6 | 0.057 |
|  | T2DM | 7 | 340/338 | -0.75 [-1.00, -0.50] | <0.001 | 60 | 0.247 |  |  |  |
| **Baseline FBG level (mmol/L)** | <7.0 | 2 | 474/472 | -0.03 [-0.24, 0.18] | 0.746 | 60 | 0.122 | 0.037 | 75.8 | 0.112 |
|  | ≥10.0 | 2 | 90/90 | -0.92 [-1.17, -0.67] | <0.001 | 0 | 0 |  |  |  |
|  | 7.0–9.9 | 5 | 250/248 | -0.67 [-1.01, -0.33] | <0.001 | 60 | 0.291 |  |  |  |
| **Age** | 45–59 | 8 | 764/760 | -0.51 [-0.80, -0.22] | <0.001 | 90 | 0.376 | 0.148 | 11.4 | 0.296 |
|  | 60–74 | 1 | 50/50 | -1.52 [-2.50, -0.54] | 0.002 | NA | NA |  |  |  |

**Supplementary Table 8.** Subgroup analysis of the effect of JLD on changes in TG

| **Subgroup** | **Group** | **No. of trials** | **No of participants** | **Effect size (MD [95% CI])** | **P** | **I² (%)** | **τ²** | **P for Interaction** | **R² (%)** | ***P_Holm*** |
| --- | --- | --- | --- | --- | --- | --- | --- | --- | --- | --- |
| **Trial duration (weeks)** | <12 | 2 | 124/124 | -0.82 [-0.99, -0.64] | <0.001 | 0 | 0.025 | 0.099 | 24.5 | 0.198 |
|  | ≥12 | 8 | 725/721 | -0.43 [-0.65, -0.21] | <0.001 | 90 | 0.265 |  |  |  |
| **Health status** | PD | 2 | 474/472 | -0.12 [-0.42, 0.19] | 0.453 | 90 | 0.204 | 0.003 | 69.7 | 0.013 |
|  | T2DM | 8 | 375/373 | -0.65 [-0.80, -0.50] | <0.001 | 40 | 0.14 |  |  |  |
| **Baseline FBG level (mmol/L)** | <7.0 | 2 | 474/472 | -0.12 [-0.42, 0.19] | 0.453 | 90 | 0.204 | 0.004 | 78.5 | 0.013 |
|  | ≥10.0 | 2 | 90/90 | -0.87 [-1.08, -0.67] | <0.001 | 0 | 0 |  |  |  |
|  | 7.0–9.9 | 6 | 285/283 | -0.58 [-0.72, -0.44] | <0.001 | 20 | 0.085 |  |  |  |
| **Age** | 45–59 | 9 | 799/795 | -0.52 [-0.75, -0.29] | <0.001 | 90 | 0.306 | 0.948 | 0 | 0.948 |
|  | 60–74 | 1 | 50/50 | -0.50 [-0.66, -0.34] | <0.001 | NA | NA |  |  |  |

**Supplementary Table 9. Meta-regression results on FBG.***

| **Variables** | **β (95% CI)** | **P-value** |
| --- | --- | --- |
| **Trial duration** |  |  |
| 12 weeks | ref | – |
| <12 weeks | −0.98 (−1.64, −0.32) | 0.004 |
| **Age** |  |  |
| <45 | ref | – |
| 45–59 | 0.42 (−0.15, 0.98) | 0.148 |
| **Baseline FBG level (mmol/L)** |  |  |
| <7.0 | ref | – |
| 7.0–9.9 | 0.57 (−0.63, 1.77) | 0.349 |
| ≥10.0 | −0.99 (−2.45, 0.48) | 0.188 |
| **Background treatment** |  |  |
| Yes | ref | – |
| No (vs Yes) | 0.96 (−0.10, 2.01) | 0.076 |
| **Health status** |  |  |
| pre-T2DM | ref | – |

*Using the restricted maximum likelihood (REML) method, the residual between-study variance was estimated (τ² = 0.0959, SE = 0.3096), with the estimated τ (the square root of τ²) being 0.3096. The degree of heterogeneity across studies was moderate, and the test for residual heterogeneity indicated significant heterogeneity remained (QE = 46.865, df = 14, p < 0.001). The model explained 81.02% of the observed heterogeneity (R²), suggesting that the majority of the variability in effect sizes could be attributed to the included moderators (trial duration, age, baseline FBG level, background treatment, and status). The overall test of moderators was statistically significant (QM = 56.498, df = 5, p < 0.001), indicating that these predictors significantly accounted for the between-study variation in effect sizes.

**Supplementary Table 10. Meta-regression results on 2hPG.***

| **Variables** | **β (95% CI)** | **P-value** |
| --- | --- | --- |
| **Trial duration** |  |  |
| 12 weeks | ref | – |
| <12 weeks | 0.76 (0.00, 1.52) | 0.051 |
| **Age** |  |  |
| <45 | ref | – |
| 45–59 | 0.58 (−0.35, 1.51) | 0.218 |
| **Baseline FBG level (mmol/L)** |  |  |
| <7.0 | ref | – |
| 7.0–9.9 | −0.08 (−1.34, 1.18) | 0.9 |
| ≥10.0 | −0.44 (−2.06, 1.18) | 0.595 |
| **Background treatment** |  |  |
| Yes | ref | – |
| **Health status** |  |  |
| PD | ref | – |

*Using the restricted maximum likelihood (REML) method, the residual between-study variance was estimated (τ² = 0.1868, SE = 0.4322), with the estimated τ (the square root of τ²) being 0.4322. The degree of heterogeneity across studies was moderate, and the test for residual heterogeneity indicated that significant heterogeneity remained (QE = 36.325, df = 12, p = 0.0003). The model explained 49.93% of the observed heterogeneity (R²), suggesting that approximately half of the variability in effect sizes could be attributed to the included moderators(trial duration, age, baseline FBG level, background treatment, and status). The overall test of moderators did not reach statistical significance (QM = 11.330, df = 6, p = 0.079), indicating that the included predictors collectively did not significantly account for between-study differences in effect sizes.

**Supplementary Table 11. Meta-regression results on HbA1c.***

| **Variables** | **β (95% CI)** | **P-value** |
| --- | --- | --- |
| **Trial duration** |  |  |
| 12 weeks | ref | – |
| <12 weeks | −0.70 (−1.26, −0.14) | 0.0138 |
| **Age** |  |  |
| <45 | ref | – |
| 45–59 | 0.39 (−0.19, 0.97) | 0.187 |
| **Baseline FBG level (mmol/L)** |  |  |
| <7.0 | ref | – |
| 7.0–9.9 | 0.05 (−0.92, 1.02) | 0.924 |
| ≥10.0 | 0.08 (−1.09, 1.26) | 0.89 |
| **Background treatment** |  |  |
| Yes | ref | – |
| No | 0.23 (−0.58, 1.03) | 0.581 |
| **Health status** |  |  |
| pre-T2DM | ref | – |

*Using the restricted maximum likelihood (REML) method, the residual between-study variance was estimated (τ² = 0.0784, SE = 0.2800), with the estimated τ (the square root of τ²) being 0.2800. The degree of heterogeneity across studies was moderate, and the test for residual heterogeneity indicated that significant heterogeneity remained. The model explained 62.98% of the observed heterogeneity (R²), suggesting that more than half of the variability in effect sizes could be attributed to the included moderators(trial duration, age, baseline FBG level, background treatment, and status). If the overall test of moderators reaches statistical significance, it would indicate that these predictors significantly account for the between-study variation in effect sizes.

**Supplementary Table 12. Meta-regression results on HOMA-IR.***

| **Variables** | **β (95% CI)** | **P-value** |
| --- | --- | --- |
| **Trial duration** |  |  |
| 12 weeks | ref | – |
| <12 weeks | −0.04 (−1.48, 1.41) | 0.961 |
| Age |  |  |
| <45 | ref | – |
| 45–59 | 0.46 (−0.52, 1.45) | 0.354 |
| **Baseline FBG level (mmol/L)** |  |  |
| <7.0 | ref | – |
| 7.0–9.9 | −0.21 (−1.41, 0.99) | 0.732 |
| ≥10.0 | −0.11 (−1.80, 1.57) | 0.894 |
| **Background treatment** |  |  |
| Yes | ref | – |
| **Health status** |  |  |
| pre-T2DM | ref | – |

*Using the restricted maximum likelihood (REML) method, the residual between-study variance was estimated (τ² = 0.2217, SE not shown), with the estimated τ (the square root of τ²) being 0.4710. The degree of heterogeneity across studies was substantial, and the test for residual heterogeneity indicated that significant heterogeneity remained (QE value not specified here). Compared with the null model (τ² = 0.1633), the model including moderators resulted in a larger residual variance, yielding a negative R² (–35.73%). This suggests that the included moderators (trial duration, age, baseline FBG level, background treatment, and status) did not effectively explain the between-study heterogeneity in effect sizes, and may have even increased unexplained variance. In other words, this meta-regression model failed to improve the explanation of between-study variability.

**Supplementary Table 13. Meta-regression results on TG.***

| **Variables** | **β (95% CI)** | **P-value** |
| --- | --- | --- |
| **Trial duration** |  |  |
| 12 weeks | ref | – |
|  | −0.04 (−1.48, 1.41) | 0.961 |
| **Age** |  |  |
|  | ref | – |
| 45–59 | 0.46 (−0.52, 1.45) | 0.354 |
| **Baseline FBG level (mmol/L)** |  |  |
|  | ref | – |
| 7.0–9.9 | −0.21 (−1.41, 0.99) | 0.732 |
| ≥10.0 | −0.11 (−1.80, 1.57) | 0.894 |
| **Background treatment** |  |  |
| Yes | ref | – |
| **Health status** |  |  |
| pre-T2DM | ref | – |

*Using the restricted maximum likelihood (REML) method, the residual between-study variance was estimated. The unadjusted model yielded τ² = 0.0406, while the model including moderators resulted in τ² = 0.0125, with the estimated τ (the square root of τ²) being 0.112. Compared with the null model, the model explained 69.18% of the observed heterogeneity (R²), suggesting that most of the variability in effect sizes could be attributed to the included moderators(trial duration, age, baseline FBG level, background treatment, and status). Although the test for residual heterogeneity indicated that significant variability remained across studies, if the overall test of moderators is statistically significant, it would confirm that these predictors effectively account for between-study differences.

**Supplementary Table 14. Sensitivity analysis excluded treatments without high-risk.**

| **Outcome** | **Number of studies** | **MD (95% CI)** | **P-value (effect size)** | **Prediction Interval** | **I² (%)** | **P-value (Q-test)** |
| --- | --- | --- | --- | --- | --- | --- |
| FBG | 16 | -0.78 [-1.05, -0.52] | <0.001 | [-1.75, 0.18] | 84.0 | <0.001 |
| 2hPG | 14 | -1.55 [-2.01, -1.10] | <0.001 | [-3.06, -0.05] | 80.7 | <0.001 |
| HbA1c | 13 | -0.82 [-1.14, -0.51] | <0.001 | [-1.94, 0.30] | 92.7 | <0.001 |
| HOMA-IR | 11 | -0.81 [-1.22, -0.39] | 0.002 | [-2.19, 0.58] | 93.5 | <0.001 |
| HDL-C | 7 | 0.20 [0.07, 0.34] | 0.01 | [-0.15, 0.56] | 93.0 | <0.001 |
| LDL-C | 7 | -0.60 [-1.01, -0.19] | 0.02 | [-1.81, 0.60] | 96.3 | <0.001 |
| TC | 7 | -0.48 [-0.86, -0.09] | 0.02 | [-1.51, 0.56] | 90.4 | <0.001 |
| TG | 8 | -0.46 [-0.72, -0.20] | 0.004 | [-1.20, 0.28] | 91.2 | <0.001 |

**Supplementary Table 15. Sensitivity analysis excluded treatments without standard background.**

| **Outcome** | **Number of studies** | **MD (95% CI)** | **P-value (effect size)** | **Prediction Interval** | **I² (%)** | **P-value (Q-test)** |
| --- | --- | --- | --- | --- | --- | --- |
| FBG | 18 | -1.10 [-1.56, -0.64] | <0.001 | [-2.48, 0.28] | 90.8 | <0.001 |
| 2hPG | 16 | -1.62 [-1.97, -1.26] | <0.001 | [-2.90, -0.35] | 88.5 | <0.001 |
| HbA1c | 15 | -0.83 [-1.08, -0.57] | <0.001 | [-1.65, 0.00] | 82.3 | <0.001 |
| HOMA-IR | 10 | -0.83 [-1.28, -0.37] | 0.003 | [-2.29, 0.64] | 94.2 | <0.001 |
| HDL-C | 6 | 0.28 [0.21, 0.35] | <0.001 | [0.10, 0.46] | 47.7 | 0.089 |
| LDL-C | 7 | -0.89 [-1.32, -0.46] | 0.002 | [-1.97, 0.19] | 83.0 | <0.001 |
| TC | 7 | -0.75 [-1.07, -0.42] | 0.001 | [-1.43, -0.07] | 57.9 | 0.027 |
| TG | 6 | 0.28 [0.21, 0.35] | <0.001 | [0.15, 0.42] | 40.9 | 0.089 |

## 2.2 Supplementary Figures

**Legend for Figures S1–S8 (Leave-one-out sensitivity analyses).**
Each line represents the pooled effect size recalculated after omitting one study at a time. The central marker (θ̂*) indicates the effect size estimated using a random-effects model, with the horizontal line showing its 95% confidence interval (CI). Changes in I² values are also displayed to reflect heterogeneity after exclusion. Stability of the pooled estimates suggests that no single study disproportionately influenced the overall results.


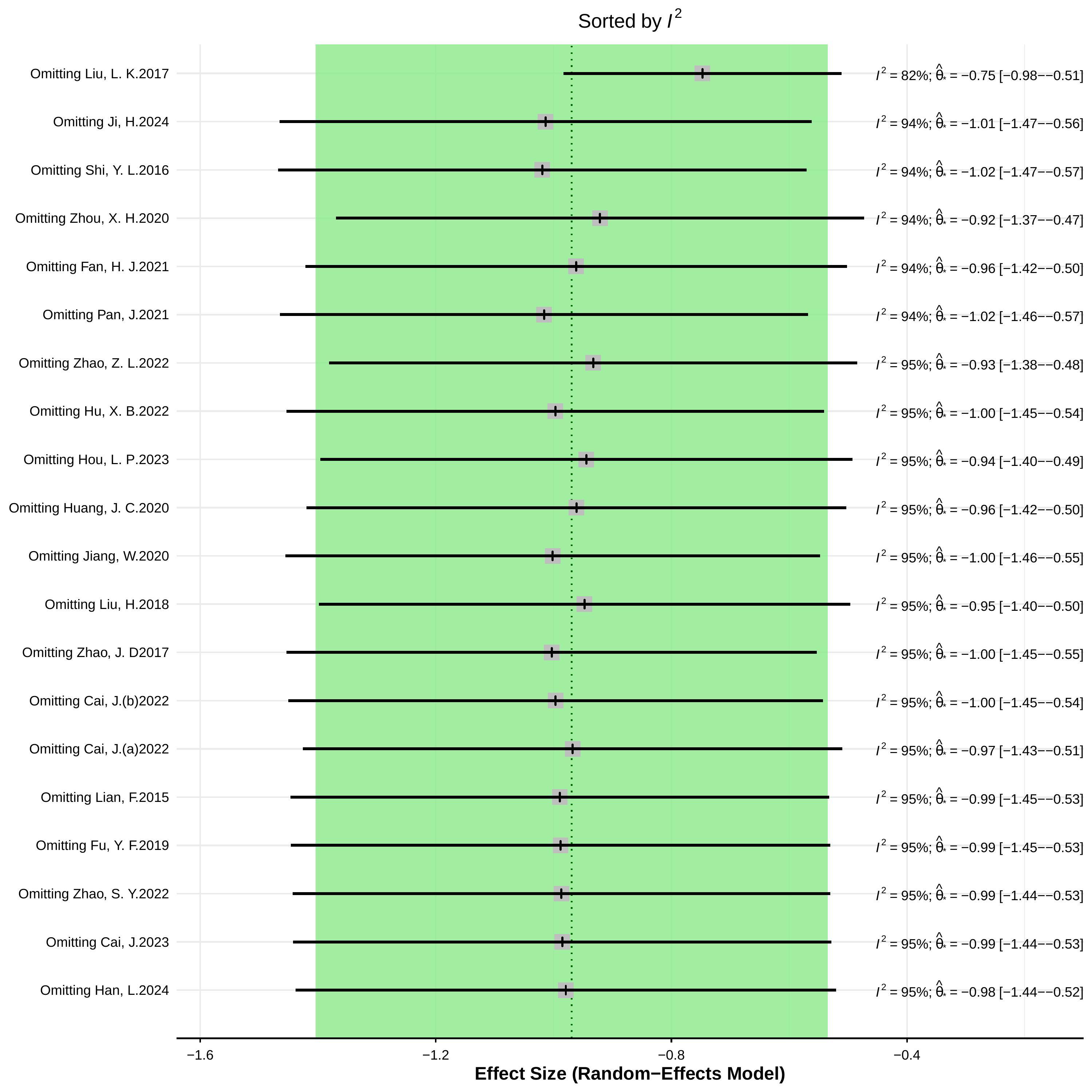


**Supplementary Figure 1.** Sensitivity analysis of the effect of JLD on FBG (leave-one-out).


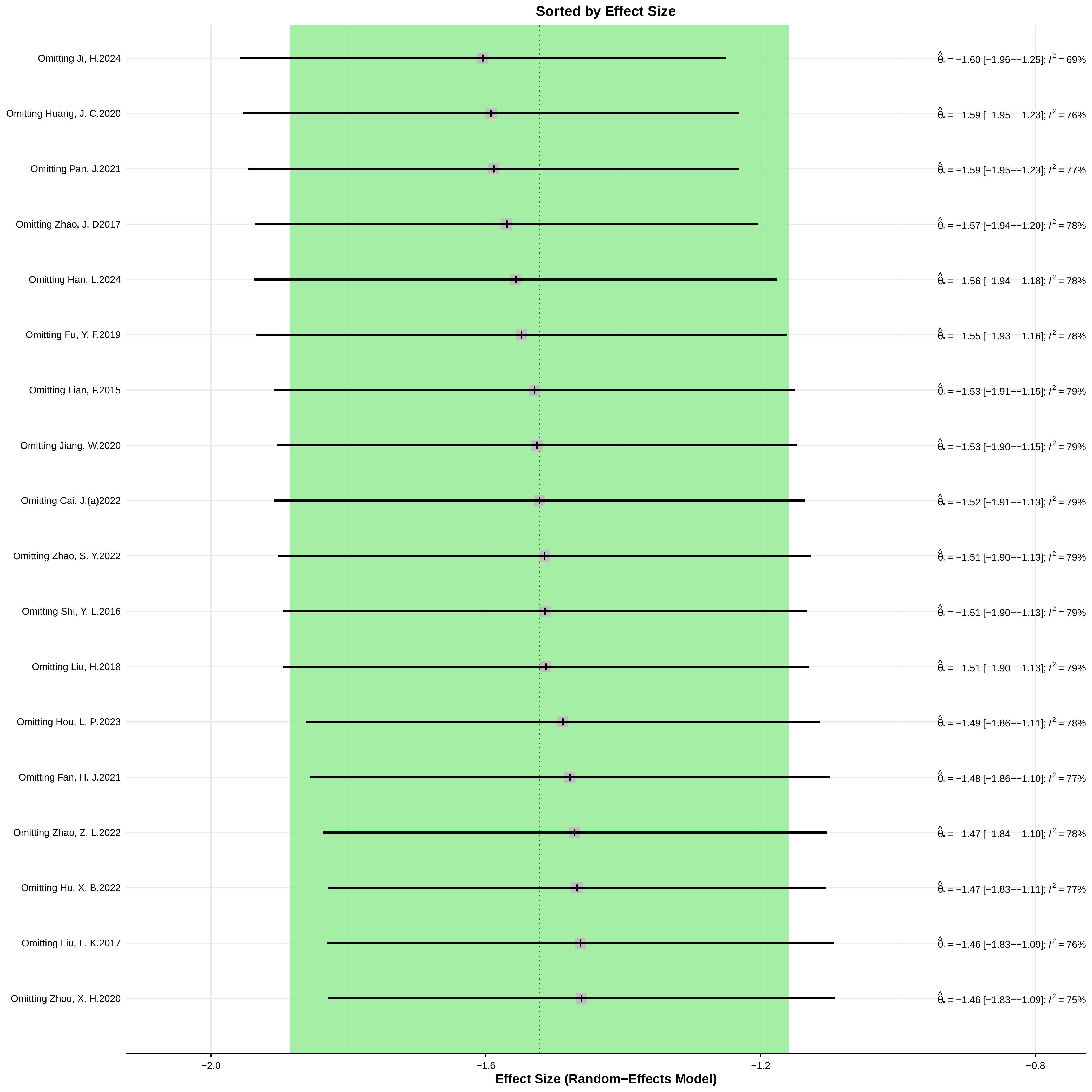


**Supplementary Figure 2.** Sensitivity analysis of the effect of JLD on 2h-PG (leave-one-out).


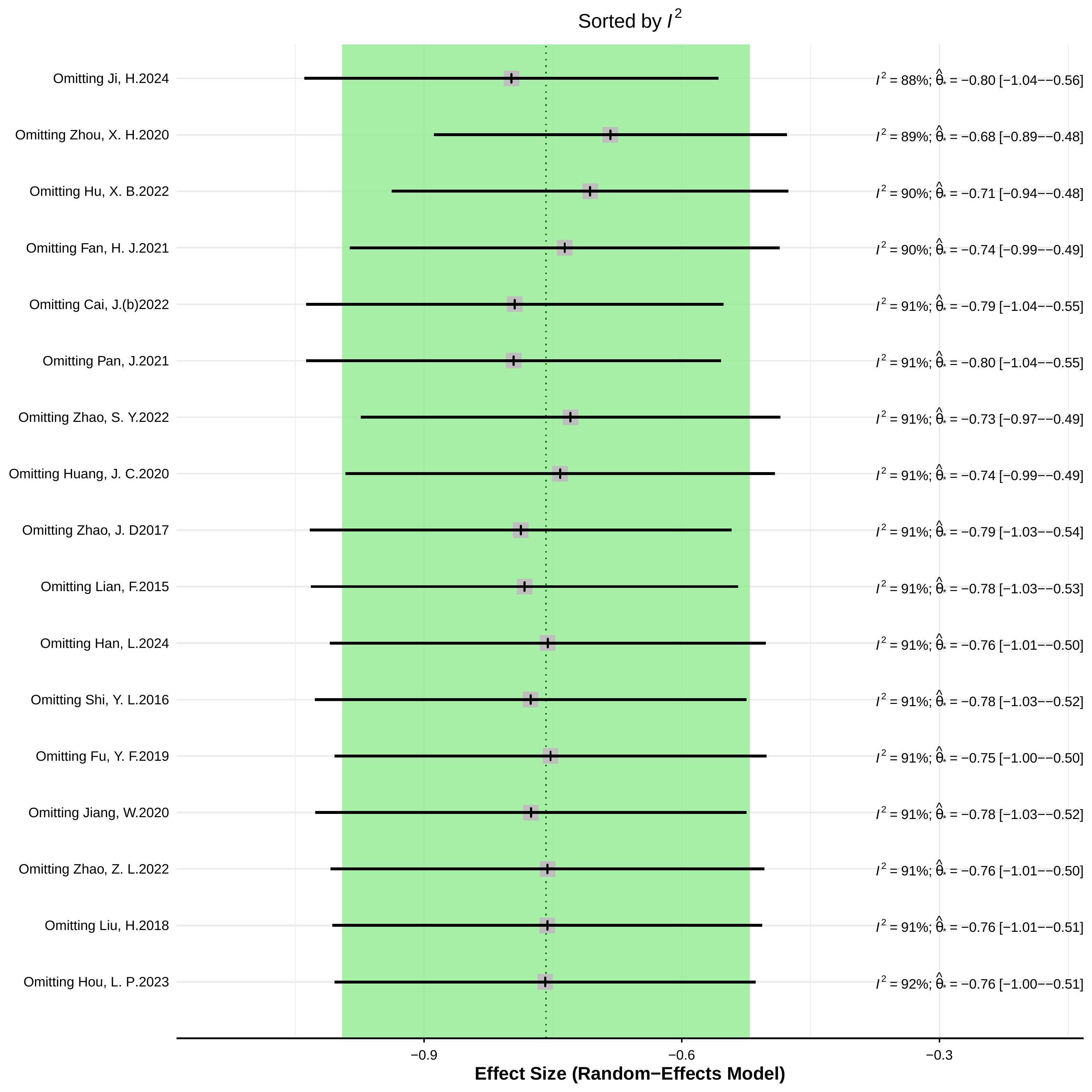
**Supplementary Figure 3.** Sensitivity analysis of the effect of JLD on HbAc (leave-one-out).


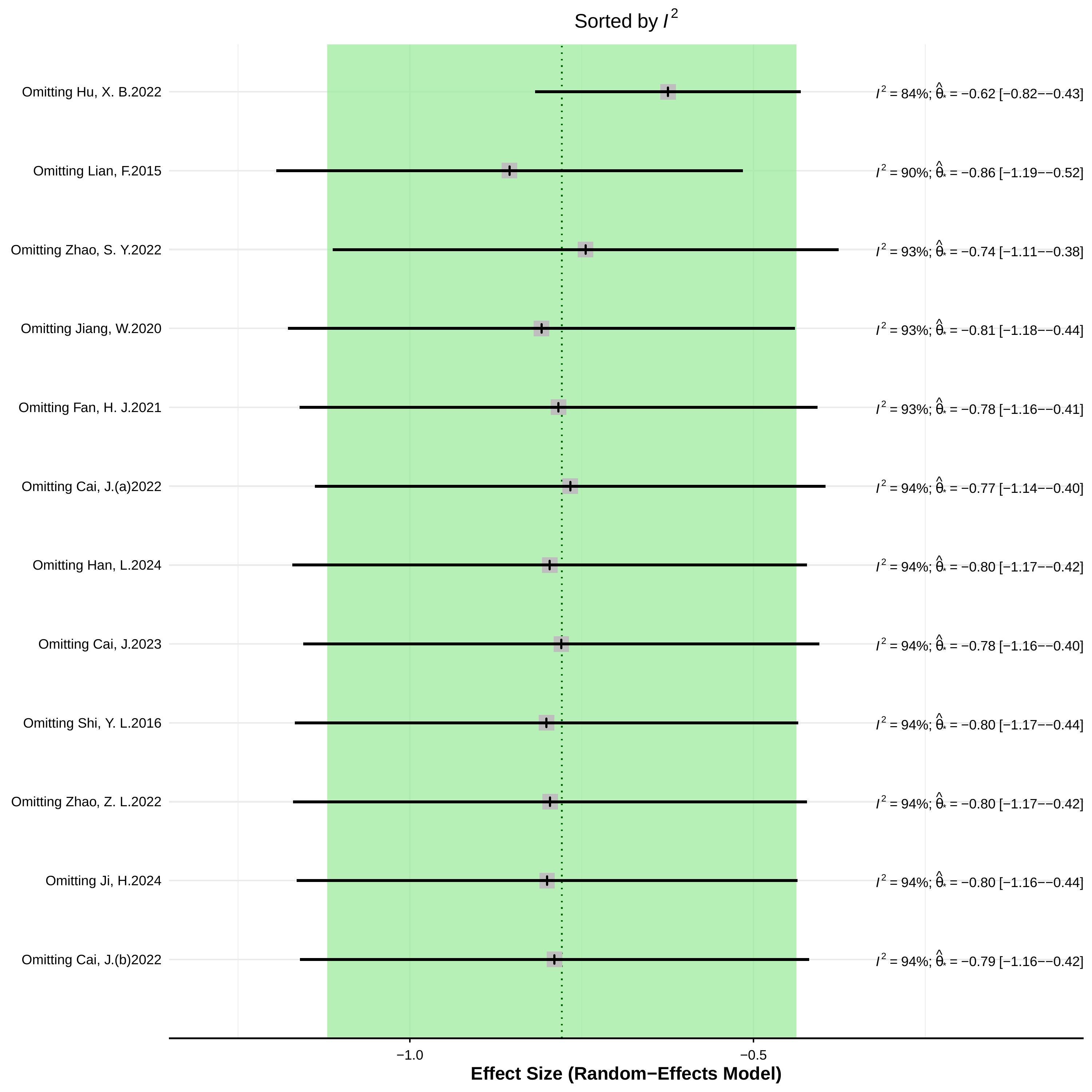


**Supplementary Figure 4.** Sensitivity analysis of the effect of JLD on HOMA-IR (leave-one-out).


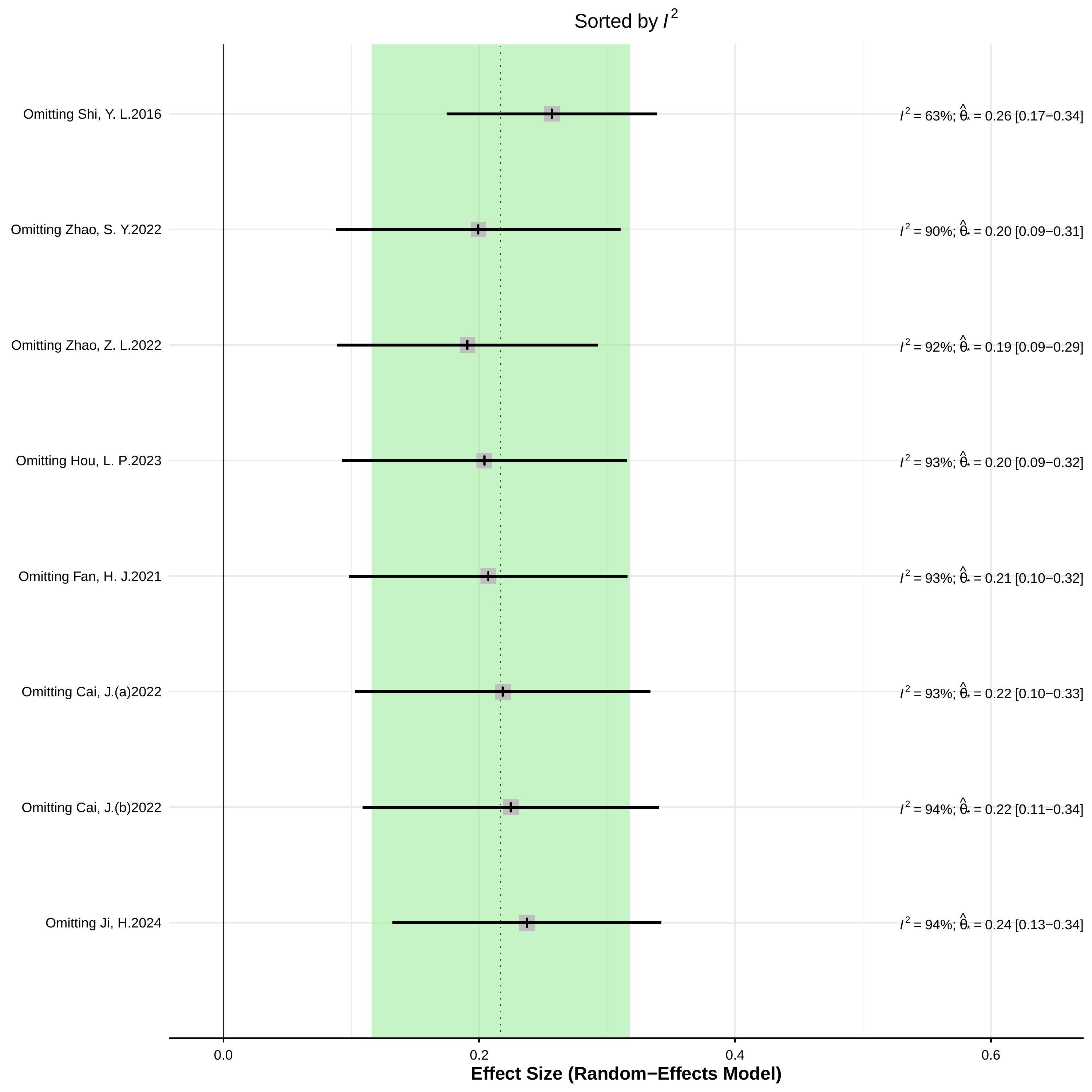


**Supplementary Figure 5.** Sensitivity analysis of the effect of JLD on HDL-C (leave-one-out).


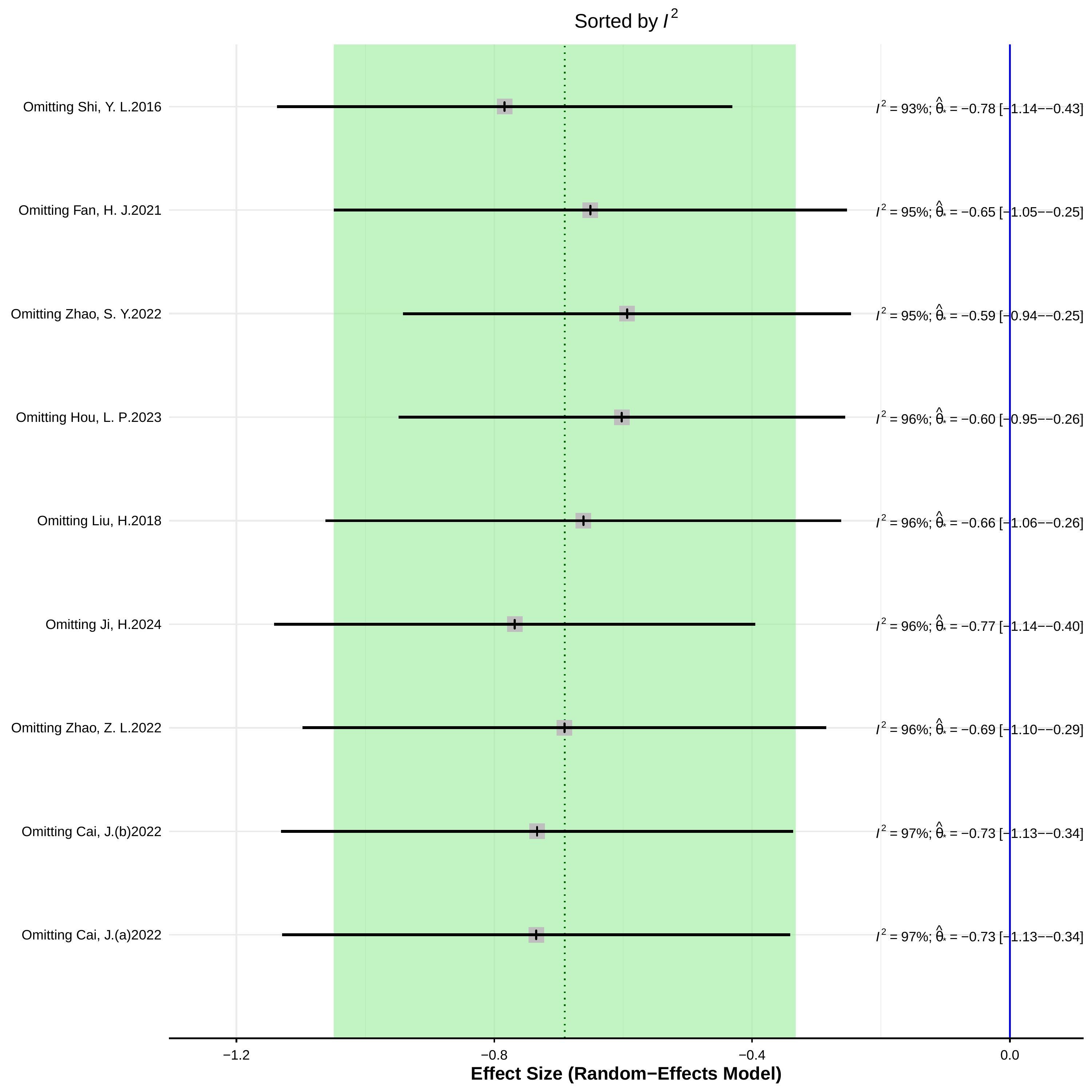


**Supplementary Figure 6.** Sensitivity analysis of the effect of JLD on LDL-C (leave-one-out).


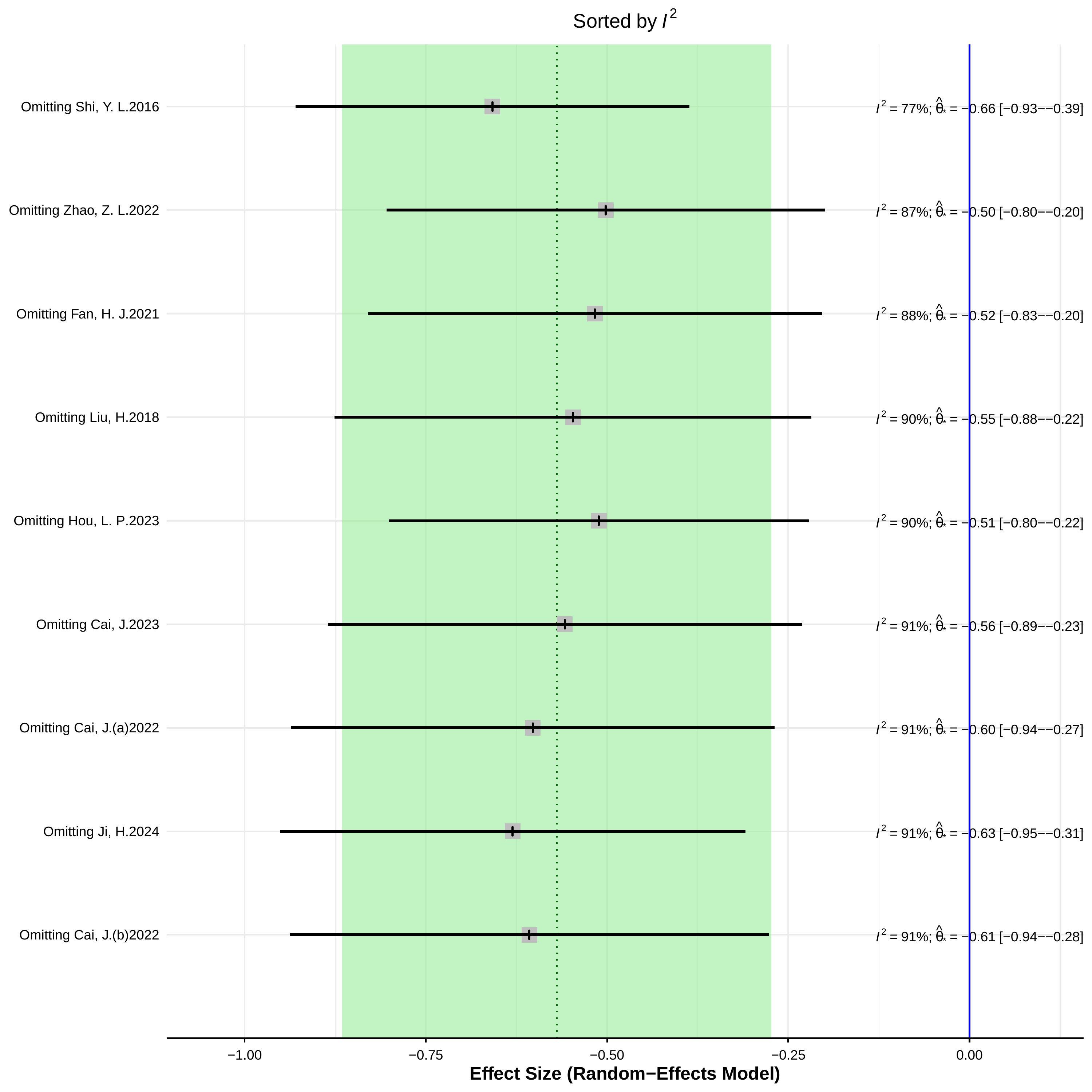


**Supplementary Figure 7.** Sensitivity analysis of the effect of JLD on TC (leave-one-out).

**
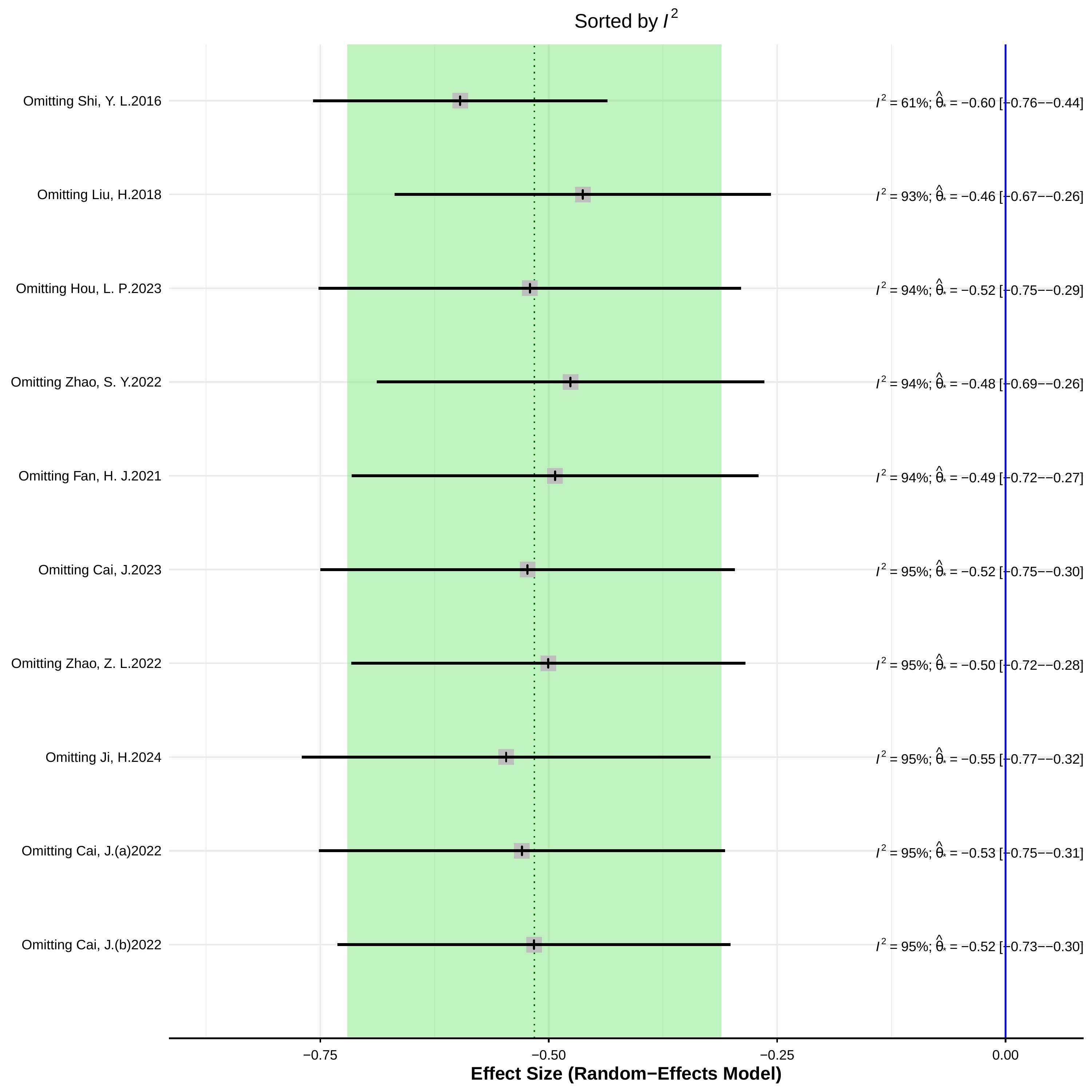
**

**Supplementary Figure 8.** Sensitivity analysis of the effect of JLD on TG (leave-one-out).

**
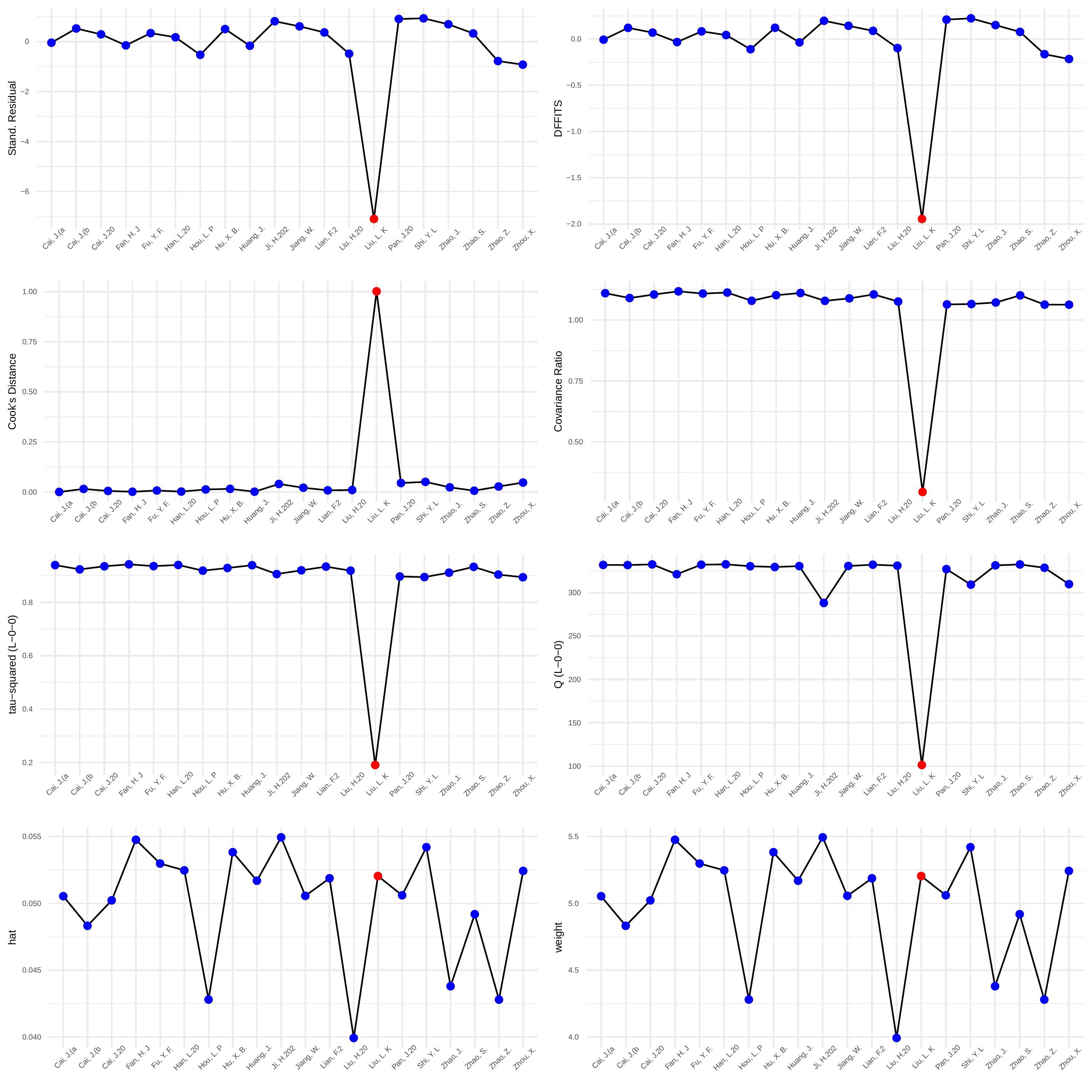
**

**Supplementary Figure 9. Residual and influence diagnostics plot for FBG**


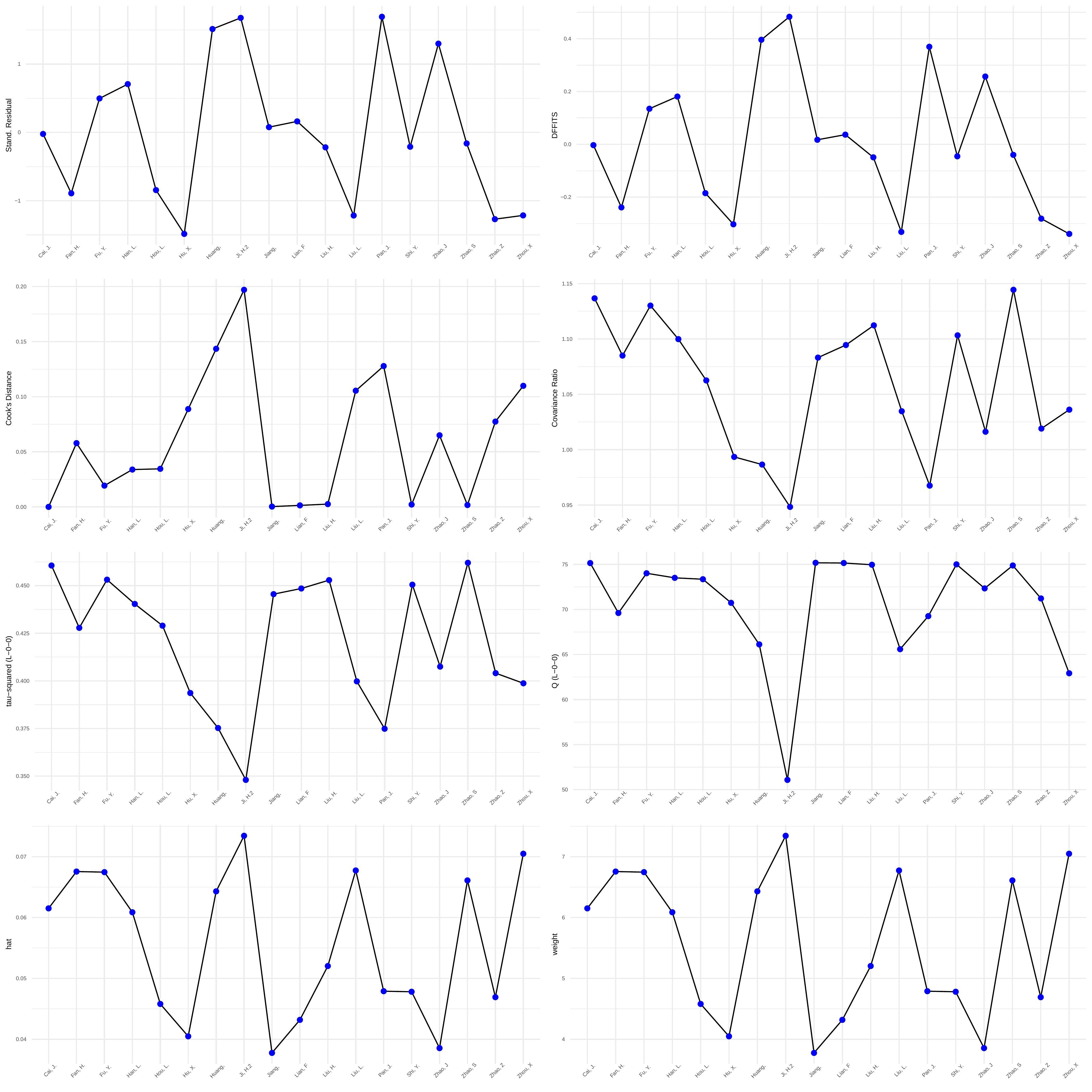


**Supplementary Figure 10. Residual and influence diagnostics plot for 2h-PG**

**
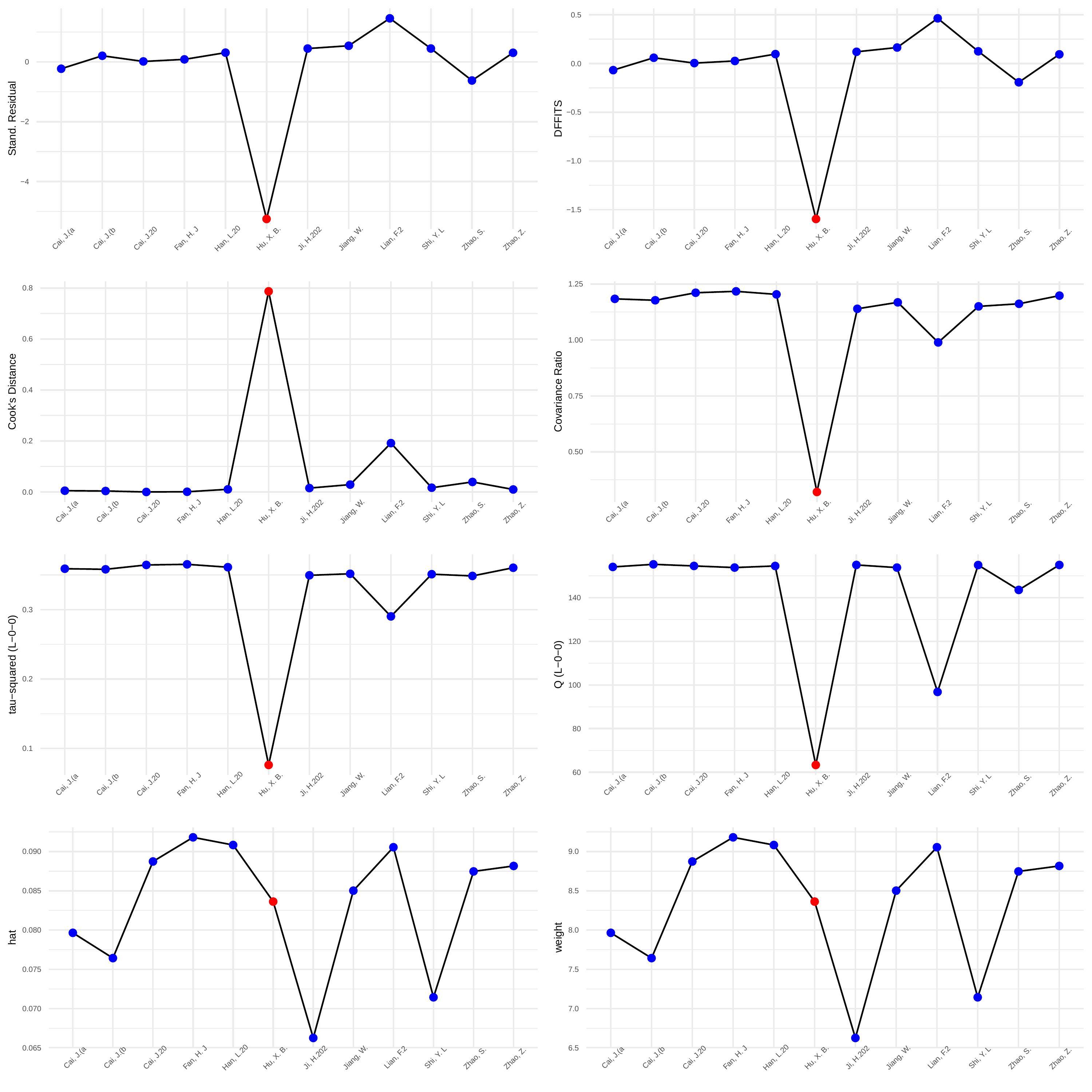
**

**Supplementary Figure 11. Residual and influence diagnostics plot for HOMA-IR**


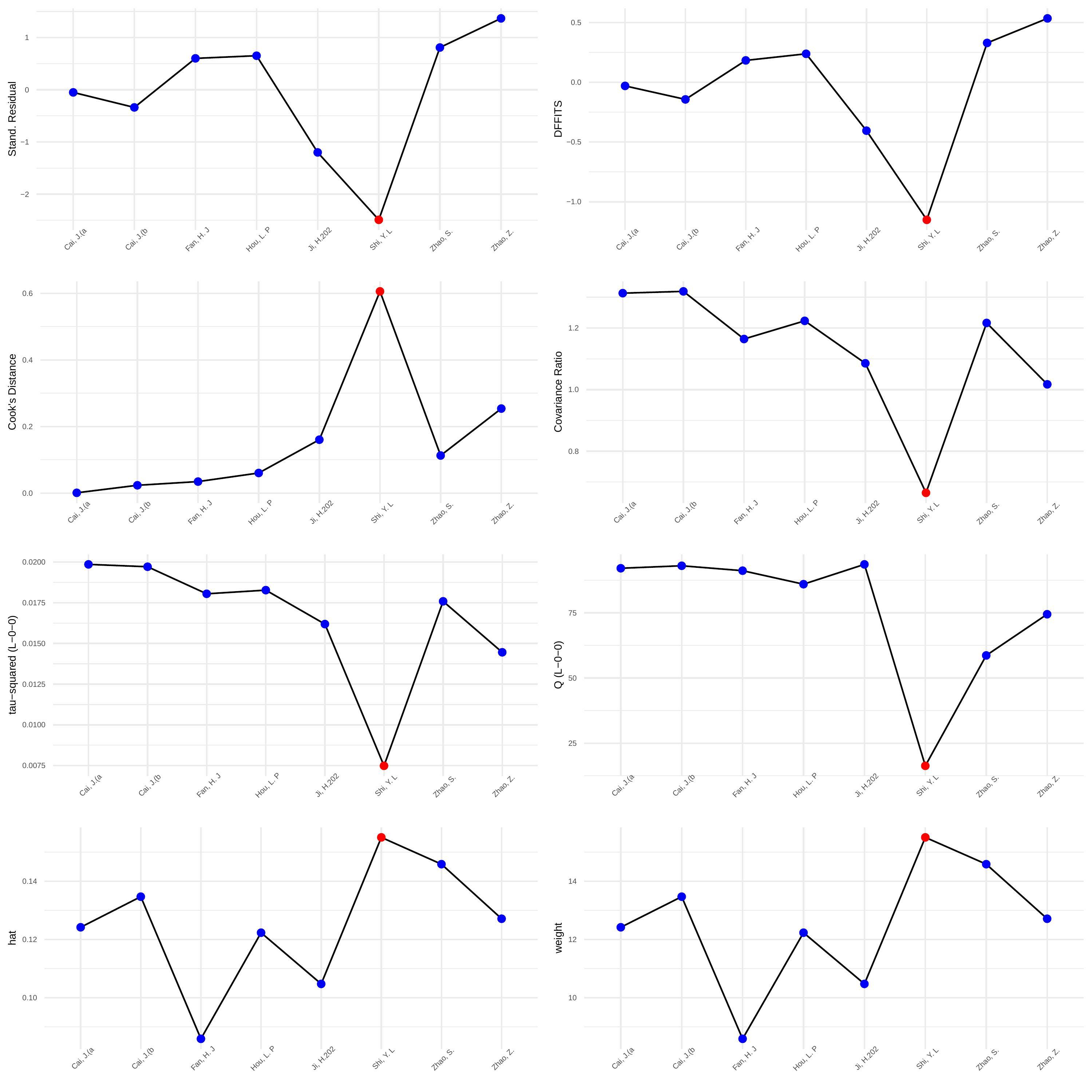

**Supplementary Figure 12. Residual and influence diagnostics plot for HDL-C**


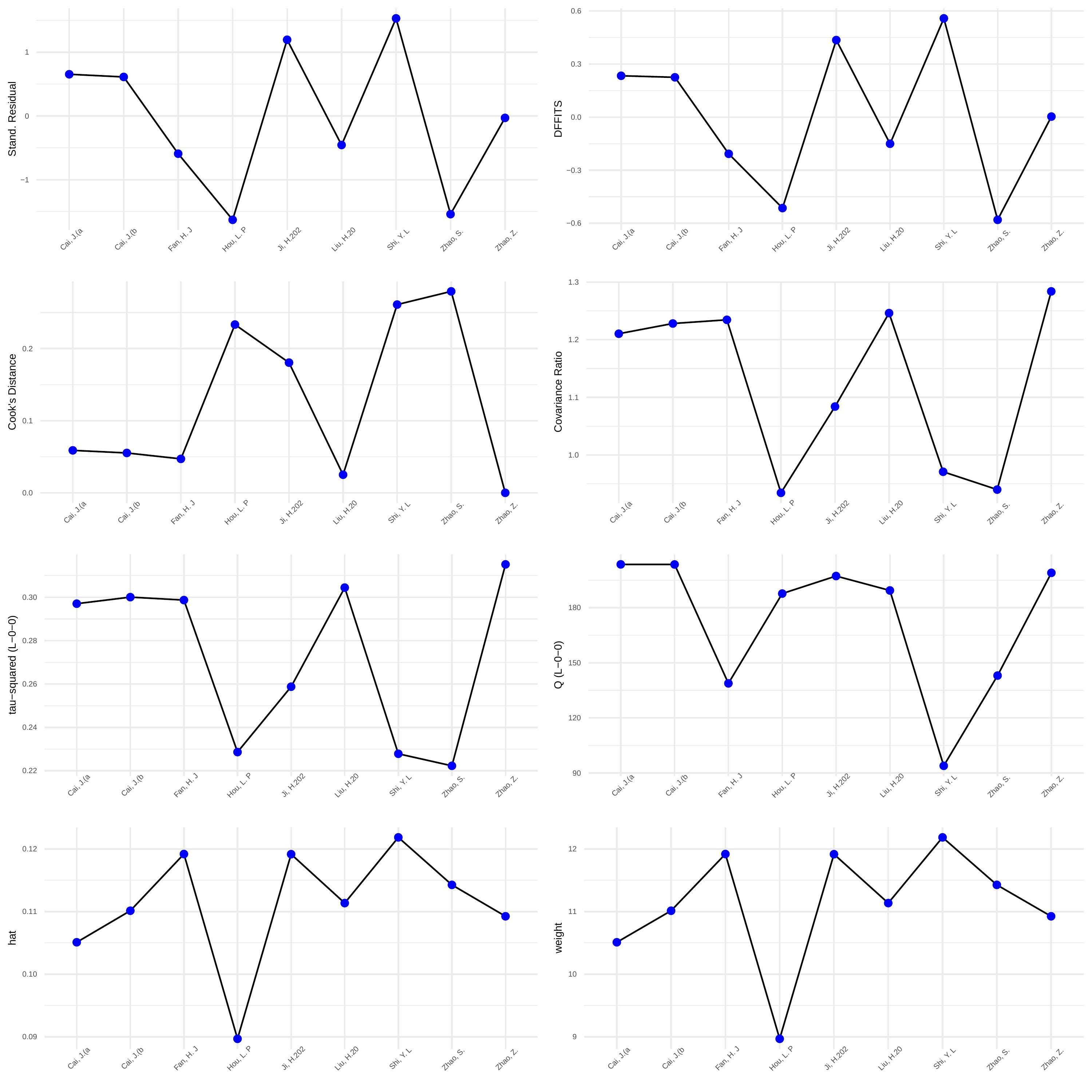


**Supplementary Figure 13. Residual and influence diagnostics plot for LDL-C**


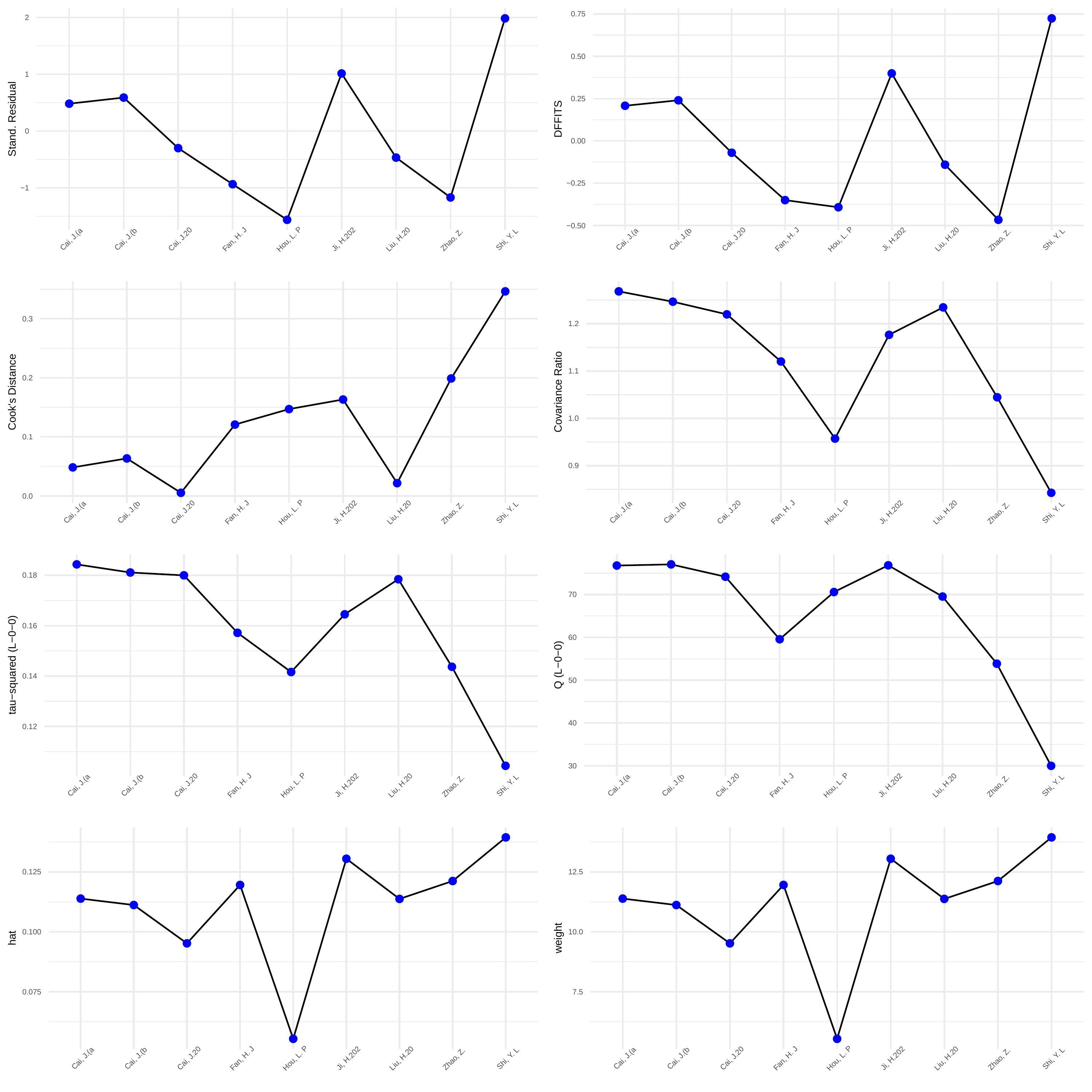
**Supplementary Figure 14. Residual and influence diagnostics plot for TC**

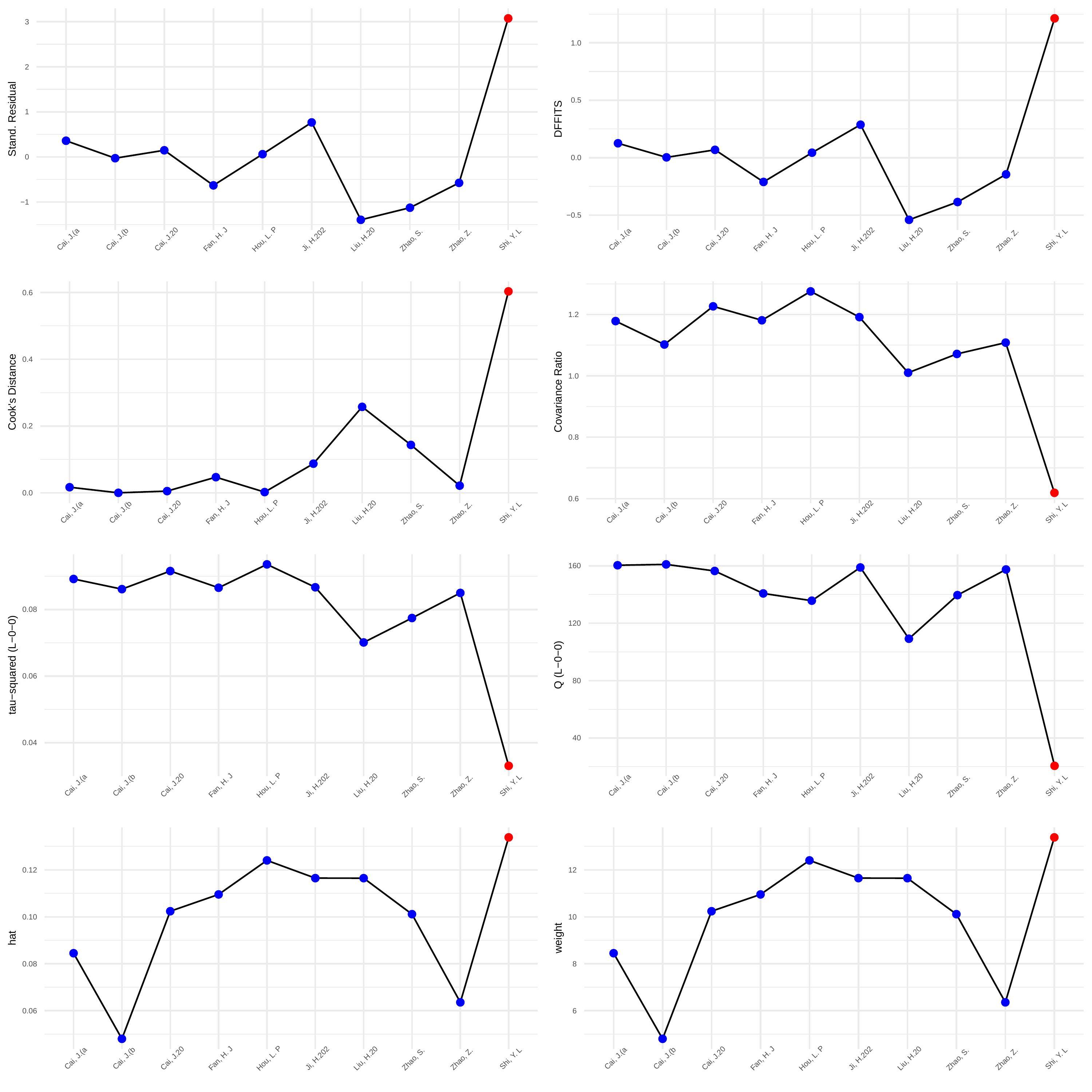


**Supplementary Figure 15. Residual and influence diagnostics plot for TG**

**
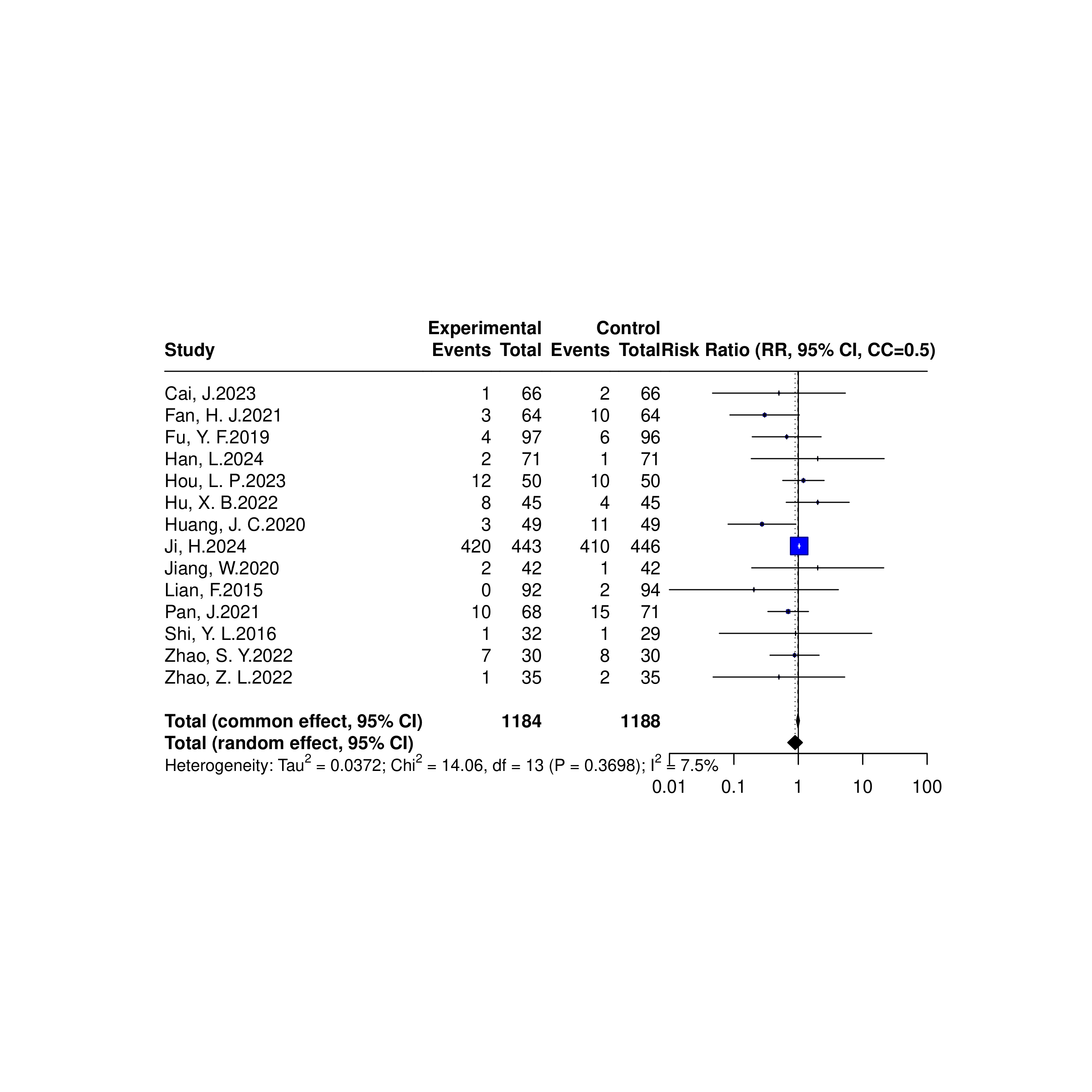
**

**Supplementary Figure 16. Sensitivity analysis applying a continuity correction of 0.5 for zero-event studies.**

**Squares indicate study-specific effect sizes with sizes proportional to study weights, horizontal lines represent 95% confidence intervals (CIs), and the diamond represents the pooled effect estimate.**
